# Coordinated Expressional Landscape of the Human Placental miRNome and Transcriptome

**DOI:** 10.3389/fcell.2021.697947

**Published:** 2021-07-21

**Authors:** Rain Inno, Triin Kikas, Kristiina Lillepea, Maris Laan

**Affiliations:** Human Genetics Research Group, Institute of Biomedicine and Translational Medicine, Faculty of Medicine, University of Tartu, Tartu, Estonia

**Keywords:** human placenta, miR-seq, gestational dynamics, pregnancy complications, preeclampsia, transcriptome (RNA-seq), miR-eQTL, genetic association study

## Abstract

Placenta is a unique organ that serves its own function, and contributes to maternal gestational adaptation and fetal development. Coordination of its transcriptome to satisfy all the maternal-fetal needs across gestation is not fully understood. MicroRNAs are powerful transcriptome modulators capable to adjust rapidly the expression level and dynamics of large gene sets. This MiR-Seq based study presents a multi-omics investigation of the human placental miRNome and its synergy with the transcriptome. The analysis included 52 placentas representing three trimesters of normal pregnancy, and term cases of late-onset preeclampsia (LO-PE), gestational diabetes and affected fetal growth. Gestational-age dependent differential expression (FDR < 0.05) was detected for 319 of 417 tested miRNAs (76.5%). A shared list of target genes of dynamic miRNAs suggested their coordinated action. The most abundant miR-143-3p revealed as a marker for pregnancy progression. The data suggested critical, but distinct roles of placenta-specific imprinted C19MC and C14MC miRNA clusters. Paternally encoded primate-specific C19MC was highly transcribed during first trimester, potentially fine-tuning the early placental transcriptome in dosage-sensitive manner. Maternally encoded eutherian C14MC showed high expression until term, underlining its key contribution across gestation. A major shift in placental miRNome (16% miRNAs) was observed in LO-PE, but not in other term pregnancy complications. Notably, 13/38 upregulated miRNAs were transcribed from C19MC and only one from C14MC, whereas 11/28 downregulated miRNAs represented C14MC and none C19MC. miR-210-3p, miR-512-5p, miR-32-5p, miR-19a-3p, miR-590-3p, miR-379-5p were differentially expressed in LO-PE and cases of small-for-gestational-age newborns, supporting a shared etiology. Expression correlation analysis with the RNA-Seq data (16,567 genes) of the same samples clustered PE-linked miRNAs into five groups. Large notable clusters of miRNA–gene pairs showing directly and inversely correlated expression dynamics suggested potential functional relationships in both scenarios. The first genome-wide study of placental miR-eQTLs identified 66 placental SNVs associated with the expression of neighboring miRNAs, including PE-linked miRNAs miR-30a-5p, miR-210-3p, miR-490-3p and miR-518-5p. This study provided a rich catalog of miRNAs for further in-depth investigations of their individual and joint effect on placental transcriptome. Several highlighted miRNAs may serve as potential biomarkers for pregnancy monitoring and targets to prevent or treat gestational disorders.

## Introduction

The placenta is a temporary mammalian organ that connects the maternal and fetal circulatory systems. Molecules produced by the placenta contribute to fetal developmental programming and support the maternal organism to cope with the pregnancy (Aplin et al., 2020). Alterations in placental gene expression may lead to its aberrant function and pregnancy complications (Sõber et al., 2015, 2016; Yong and Chan, 2020; Kikas et al., 2021).

MicroRNAs (abbreviated as miRNAs) are critical modulators of post-transcriptional levels of mRNAs, fine-tuning the composition of cellular proteome. Upon binding to 3′UTRs of mRNA transcripts, miRNAs guide their target to degradation or temporary translational inhibition. The transcript level of each mRNA is modulated by several jointly acting miRNAs and each miRNA contributes to fine-tuning the expression level of hundreds or even thousands of genes. The roles of several miRNAs (miR-155, miR-210-3p, miR-518b-3p) are well known in placental function, trophoblast growth and proliferation (Chiofalo et al., 2017; Apicella et al., 2019; Ghafouri-Fard et al., 2020). Increased expression of some specific miRNAs, such as miR-210 reflect placental distress in hypoxic or other malfunctioning conditions (Pineles et al., 2007; Zhang et al., 2012). Notably, there are large imprinted clusters of miRNA genes, C19MC (Chr. 19; 46 miRNA genes; only maternal allelic expressed) and C14MC (Chr. 14; 52 miRNA genes, only paternal allelic expression) that are nearly exclusively expressed in the placenta. C19MC has evolved in the primate lineage and C14MC among eutherians (Morales-Prieto et al., 2013; Tamaru et al., 2020). C14MC is one of the largest mammalian miRNA clusters and is located within an imprinted chromosomal region DLK1-DIO3, harboring also either paternally or maternally imprinted genes (*DLK1, RTL1, MEG3*, *MEG8* and *DIO3*) and C/D small nucleolar RNAs (SNORDs) (Seitz et al., 2004; Pilvar et al., 2019). A further co-regulator of placental transcriptome is the miR-371–373 gene cluster with also restricted expression to trophoblast lineage and embryonic stem cells (Chr.19; four miRNA genes) (Wu et al., 2014). Despite the potential important role of miRNAs in shaping the placental transcriptome throughout gestation, there is limited data on how the level of individual miRNAs as well as the whole placental miRNome is correlated with the placental transcriptome (Awamleh et al., 2019; Kennedy et al., 2020).

Single nucleotide variants (SNVs) that regulate the transcriptional activity of adjacent genes are termed as Expression Quantitative Trait Loci (eQTLs) (GTEx Consortium, 2015; The GTEx Consortium, 2020). Placental eQTLs and their potential functional link to pregnancy traits have only recently gained attention (Kikas et al., 2020, 2021). Unlike protein-encoding genes, there is no published data on SNVs regulating the expression levels of miRNAs in placenta, referred as miRNA eQTLs (miR-eQTLs).

The current study aimed at comprehensive profiling of the human placental miRNome and its expression dynamics. More specifically, miR-Seq datasets of 52 placentas were analyzed for differential expression between the three trimesters of pregnancy, as well as between cases with late gestational complications compared to uneventful pregnancies at term. The functional effect of 66 differentially expressed miRNAs (DEmiRs) in preeclamptic placentas was explored using expressional correlation analysis with the corresponding RNA-Seq based transcriptome dataset. For the first time, a genome-wide approach was applied to map placental miR-eQTLs and to investigate their link to pregnancy outcomes.

## Materials and Methods

### Ethics Statement

The study utilized samples from Estonian REPROMETA (full study name “REPROgrammed fetal and/or maternal METAbolism”; recruitment 2006–2011) and HAPPY PREGNANCY (full: “Development of novel non-invasive biomarkers for fertility and healthy pregnancy”; 2013–2015) data sets. Both studies were approved by the Ethics Review Committee of Human Research of the University of Tartu, Estonia (Permissions No 146/18, 27.02.2006; 150/33, 18.06.2006; 158/80, 26.03.2007; 221/T-6, 17.12.2012; 286/M-18, 15.10.2018). All study participants were recruited, and the study material was collected at the Women’s Clinic of Tartu University Hospital, Estonia. Written informed consent to participate in the study was obtained from each individual prior to recruitment. The study was carried out in compliance with the Helsinki Declaration and all methods were carried out in accordance with approved guidelines. All participants were of white European ancestry and living in Estonia.

### Samples Utilized for the Placental miRNome Analysis

Genome-wide profiling of placental miRNome was performed for 52 placental samples representing first (*n* = 5) or second (*n* = 7) trimester of gestation, and term pregnancy (*n* = 40) (Table 1). Term placental samples were drawn before or shortly after delivery during the REPROMETA study (Supplementary Methods). The analyzed 40 term pregnancy cases (delivery after 37th gestational week, g.week) represented normal gestation, preeclampsia (PE), gestational diabetes (GD), and delivery of a small- or large-for-gestational-age newborn (SGA and LGA, newborns <10th or >90th birth weight centile, respectively). Each clinical subgroup included eight cases, matched for the gestational age, delivery mode and proportions of male/female newborns. First and second trimester placental samples had been collected from women who underwent elective surgical termination of pregnancy or medically induced abortion due to maternal medical risks. In all analyzed cases, fetal anomalies and gross chromosomal aberrations were excluded. The definition of clinical subgroups, details of collection and processing of placental samples, and DNA and RNA extraction protocols are provided in Supplementary Methods and recent publications (Sõber et al., 2015; Reiman et al., 2017; Kikas et al., 2019, 2020).

**TABLE 1:** Clinical characteristics of the pregnancies profiled for the placental miRNome.

	Early pregnancy		Term pregnancy					
Pregnancy related parameters (units)/	I trimester	II trimester	All samples	Healthy	Preeclampsia	Gestational diabetes	SGA at birth	LGA at birth
Sample size (*n*)	5	7	40	8	8	8	8	8
Maternal age (years)	24 (19–33)	24 (15–39)	29 (18–39)	33 (18–37)	27 (19–39)	33 (22–36)	25 (20–32)	30 (18–39)
Maternal height (cm)	161 (160–165)	170 (160–173)	166 (153–179)	165 (158–175)	170 (163–173)	167 (158–175)	166 (153–172)	167 (160–179)
Pre-pregnancy BMI (kg/m^2^)	21 (20–26)	22 (17–25)	24 (16–43)	24 (17–30)	26 (20–34)	26 (18–43)	21 (17–24)	24 (19–31)
Nulliparity (*n*, %)	1 (20%)	5 (65.5%)	21 (52.5%)	3 (37.5%)	6 (75%)	3 (37.5%)	7 (87.5%)	2 (25%)
Smokers (*n*)	4	Unknown	7 (17.5%)	2 (25%)	2 (25%)	1 (12.5%)	2 (25%)	0
Gestational age at birth/abortion (days)	60 (51–81)	121 (108–140)	274 (260–291)	284 (260–291)	266 (260–271)	276 (268–284)	271 (264–289)	281 (275–288)
Vaginal/CS delivery	n.a	n.a	19/21	5/3	2/6	3/5	6/2	3/5
Fetal sex (M/F)	2/3	4/3	19/21	5/3	4/4	3/5	3/5	4/4
Birth weight (g)	n.a	n.a	3756 (2004–4986)	3756 (3102–4220)	2803 (2170–3570)	4284 (3940–4680)	2517 (2004–2698)	4744 (4420–4986)
Birth length (cm)	n.a	n.a	51 (45–55)	51 (49–55)	48 (45–49)	53 (51–54)	46 (45–48)	53 (52–55)
Birth head circumference (cm)	n.a	n.a	35 (32–39)	36 (33–36)	34 (32–36)	36 (34–38)	32 (32–34)	38 (37–38)
Birth chest circumference (cm)	n.a	n.a	35 (28–39)	35 (33.5–38)	31 (28.5–35)	36 (34–38)	31 (28–34)	37 (36–39)
Placental weight (g)	n.a	n.a	545 (200–1060)	575 (420–770)	463 (340–720)	588 (500–1060)	420 (200–470)	818 (610–970)

*Data are represented as medians with ranges, except where indicated differently. Nulliparity refers to no previous childbirth. Birth data apply to the newborn. BMI, body mass index; CS, C-section; LGA, large-for-gestational-age; SGA, small-for-gestational-age; n.a., not applicable.*

### Small-RNA Sequencing and Data Processing

Initial small-RNA libraries were prepared from 1 μg total RNA (TruSeq Small RNA kit, Illumina), followed by miRNA enrichment (Caliper LabChipXT, PerkinElmer) according to manufacturer’s protocols. Small RNA-Seq libraries were sequenced on Illumina HiSeq 2000. Library preparation and sequencing were conducted in FIMM Sequencing Laboratory, University of Helsinki, Finland. Quality control of the raw reads was performed using FastQC (ver. 0.11.7) and MultiQC (ver. 1.7) (Ewels et al., 2016). Trimmomatic (ver. 0.38) was implemented to remove adapters and trim the quality of reads with the following settings - ILLUMINACLIP:2:30:9, LEADING:3, CROP:50, TRAILING:3, SLIDINGWINDOW:4:20, MINLEN:16. Reads were aligned to human genome reference GRCh38 using bowtie (ver. 1.2.2, settings: -n 1 -l 20 -q -m 40 -k 1 -t –best –strata) (Langmead et al., 2009). miRNA quantification was performed using featureCounts from the Rsubread package (ver. 1.20.6) (Liao et al., 2019) for R with miRNA annotations from miRBase 22.1 as reference (Kozomara et al., 2019).

### Bioinformatic Analysis of the Placental miR-Seq Dataset

From 2,652 quantified placental miRNAs only those with median raw read counts over 50 across all analyzed samples (*n* = 417; 15.7%) were included in statistical analyses. All subsequent computational profiling and differential expression analyses were implemented using read counts normalized with DESeq2 (ver. 1.22.2) package for R with default settings (Love et al., 2014; Supplementary Table 1). In addition, counts per million reads mapped (CPM) were quantified for the graphical presentation of miRNA expression in subgroups. False-discovery rate (FDR) in differential expression tests was applied according to Benjamini and Hochberg (1995). Test results with FDR *P* < 0.05 were considered as statistically significant. Placental miRNome was compared for the following subgroups: first (*n* = 5) vs. second trimester (*n* = 7), second trimester vs. normal term (*n* = 8) pregnancy; late gestational complications (PE, GD, SGA, LGA) vs. normal term pregnancy (each group *n* = 8); term pregnancy 46, XX (*n* = 21) vs. 46, XY (*n* = 19). Comparisons of miRNA expression between the three trimesters of gestation were carried out without and with adjustment for sex as cofactor. miRTarBase database (Huang et al., 2020) was used to assemble the lists of experimentally validated target genes for differentially expressed miRNAs (DEmiRs) across normal pregnancy, and for miRNAs encoded by the C19MC and C14MC clusters. Only target genes with high confidence were considered for the gene enrichment analysis (see below). Expression correlations of miR-143-3p, miR-92a-3p, miR-26a-5p, as well as C19MC and C14MC microRNAs with high confidence target genes were calculated using Kendall correlation coefficient (parameter tau).

mRNA/lincRNA expression data was derived from the RNA-Seq datasets that had been previously generated for the same placental samples as utilized for miR-Seq. Placental RNA-seq library preparation, sequencing and raw data processing are detailed in Supplementary Methods and in previous studies (Sõber et al., 2015, 2016; Reiman et al., 2017; Pilvar et al., 2019).

Analysis of inter-relatedness between the expression of miRNAs and mRNA/lincRNA genes in 40 term placentas also utilized the above-mentioned published RNA-Seq data. Expressional correlation of miRNA/mRNA transcripts was evaluated using Spearman’s correlation coefficient (parameter rho). Correlation analysis included 66 miRNAs showing differential expression in PE in the miR-Seq dataset and 16,567 genes with raw median read counts >50 in the RNA-Seq dataset. Spearman’s rho values for 1,093,422 miRNA-gene pairs were estimated in R and visualized as a heatmap, using R package heatmap.2 (Gregory et al., 2015). Lists of genes showing confident expressional correlation with miRNA hierarchical cluster groups G1-G5 were formed using the following criteria: median Spearman’s rho across 40 term placentas <−0.3 and for individual samples <−0.1 (negatively correlated genes); or median rho > 0.3 and for individual samples higher than rho > 0.1 (positively correlated genes). These gene lists were used as input for the gene enrichment analysis for *in silico* functional profiling.

All gene enrichment analyses based on miRNA hierarchical cluster groups were implemented in g:Profiler (ver. 1760) with default settings (Reimand et al., 2016). The recommendation for a more conservative analysis to compute functional enrichment in a custom gene list instead of all human genes in Ensembl database was followed to avoid overestimating statistically significant results (Reimand et al., 2016; Raudvere et al., 2019).

### miR-eQTL Analysis

To avoid potential confounding effect of gestational expression dynamics, the discovery analysis of placental miR-eQTLs included only term placental samples (*n* = 40). SNV genotypes were derived from the previously published genome-wide genotyping dataset of the same placental samples [Illumina HumanOmniExpress-12-v1 BeadChip (>733,000 SNVs; median spacing 2.1 kb)] (Kasak et al., 2015; Pilvar et al., 2019). The analysis was targeted to ±100 kb window extending to both directions from the start and end of miRNA genes, annotated based on miRBase (ver. 22.1). The genomic regions flanking the analyzed 417 miRNAs included 6,274 common SNVs (MAF > 0.1). In total, 17,302 linear regression association tests were carried out between SNV genotypes and miRNA expression levels, quantified as normalized miRNA read counts. All tests with miR-eQTLs were implemented in PLINK v1.07 using fetal sex and gestational age as cofactors (Purcell et al., 2007). The results were corrected for multiple testing using the Benjamini–Hochberg method, with cut-off FDR < 0.05. All of the miR-eQTLs were tested for Hardy–Weinberg equilibrium (Supplementary Table 2).

### Cohorts for the Genetic Association Testing Between miR-eQTLs and Term Pregnancy Traits

The REPROMETA study recruited 366 pregnant couples before or shortly after delivery of a singleton newborn at term (Supplementary Table 3). The cases represented pregnancies with uncomplicated gestation, PE, GD, SGA or LGA. Maternal and newborn clinical and epidemiologic data were documented retrospectively from self-reported questionnaires and medical records, collected biological materials included placental tissue (available for 326 cases) and parental blood samples.

The HAPPY PREGNANCY study recruited prospectively 2,334 pregnant women during their first antenatal visit. Longitudinal clinical and epidemiological data covers reproductive history, parental lifestyle, the course and outcome of pregnancy. The collected biological material included placental tissue (available for 1,772 cases), maternal blood and urine samples.

In both studies, the diagnosis of PE and GD followed the international guidelines at the time of recruitment (Metzger, 2010; American College of Obstetricians and Task Force on Hypertension in Pregnancy, 2013). Fetal growth was evaluated using the gestational age and sex adjusted weight centiles based on the Estonian Medical Birth Registry data (Sildver et al., 2015). Details of the REPROMETA and HAPPY PREGNANCY studies are provided in Supplementary Methods and in recent publications (Kikas et al., 2019, 2020).

### Genetical Association Testing Between miR-eQTLs and Pregnancy Traits

Genetic association testing between the identified miR-eQTLs and pregnancy traits (placental weight; newborn’s weight and height, head and chest circumference; diagnosis of PE or GD) was carried out using either linear or logistic regression (additive model) adjusted for fetal sex and gestational age as cofactors. Results were corrected for multiple testing using the Benjamini-Hochberg method, with cut-off FDR < 0.05. Initially, all miR-eQTLs were tested in the discovery dataset (*n* = 40, Table 1). Three SNVs (rs12985296, rs7046565, and rs12420868) were further analyzed in the REPROMETA (*n* = 326) and HAPPY PREGNANCY (*n* = 1,772) samples, including all cases with available placental tissue for genotyping (Supplementary Table 3).

Genotypes were generated with the TaqMan SNP Genotyping Assays (Supplementary Methods and Supplementary Table 4) using recommended experimental conditions (Applied Biosystems, Life Technologies). All association tests were carried out in PLINK v1.07 using (Purcell et al., 2007). Meta-analysis of REPROMETA and HAPPY PREGNANCY datasets was implemented in R package meta (ver. 4.15-1) (Balduzzi et al., 2019), under fixed-effect model.

## Results

### Highly Variable Expression Levels of Placental miRNAs

The dataset of 52 placental miRNomes was generated for the samples collected from first and second trimester and term pregnancy cases (*n* = 5, 7 and 40, respectively; Table 1). All subsequent analyses included 417 of total 2,652 identified miRNAs (15.7%, Table 2A), filtered for transcript levels that allow confident statistical testing (median raw read counts >50 across analyzed samples; Supplementary Table 1).

**TABLE 2:** General profile and expression dynamics of placental miRNAs.

	miRNA categories				
Category	All miRNAs	C19MC^a^ chr19q13.42	C14MC^b^ chr14q32.31	miR-371–373^c^ chr19q13.42	Other known or detected miRNAs
**(A) Comparative general profile of miRNA categories**					

Gene cluster size (kb)	n.a.	∼100 kb	∼250 kb	∼1.1 kb	n.a.
Placenta-specific	n.a.	All	All	All	n.a.
Parent of origin expression	Most biallelic	Paternal	Maternal	Unknown	Most biallelic
All miRNA genes^d^ (*n*)	1,792	46	52	4	1,690
All mature miRNA transcripts^d^ (*n*)	2,656	67	94	8	2,487
All identified placental mature miRNA transcripts in this study (*n*)	2,652	67	93	8	2,484
Placental mature miRNA transcripts with adequate expression level for confident statistical testing (*n*)^e^	417	65	58	2	292

**(B) Expressional patterns from first to second trimester – from second trimester to term pregnancy (miRNA mature transcripts: *n*, %)^e,f^**					

Down – Down	30 (7.2%)	2 (3.1%)	8 (13.8%)	0 (0%)	20 (6.8%)
Down – No change	67 **(16.1%)**	21 **(32.3%)**	1 (1.7%)	2 **(100%)**	**43 (14.7%)**
Down – Up	28 (6.7%)	14 **(21.5%)**	0 (0%)	0 (0%)	14 (4.8%)
Up – Up	35 (8.4%)	0 (0%)	0 (0%)	0 (0%)	**35 (12.0%)**
Up – No change	41 (9.8%)	0 (0%)	7 (12.1%)	0 (0%)	34 (11.6%)
Up – Down	26 (6.2%)	0 (0%)	11 **(19.0%)**	0 (0%)	15 (5.1%)
No change – Down	54 **(13.0%)**	2 (3.1%)	26 **(44.8%)**	0 (0%)	26 (8.9%)
No change – Up	38 (9.1%)	4 (6.2%)	0 (0%)	0 (0%)	34 (11.6%)
No change – No change	98 **(23.5%)**	22 **(33.8%)**	5 (8.6%)	0 (0%)	**71 (24.3%)**

*^a^Primate-specific miRNA cluster. ^b^Eutherian-specific miRNA cluster. ^c^Homologous with the mouse miR-290–295 cluster (Wu et al., 2014). ^d^Data from miRBase version 22.1 (Kozomara et al., 2019). ^e^Median raw read counts over 50 across all analyzed samples; empirically determined transcript level for robust differential expression testing. ^f^Major patterns of expression dynamics are highlighted in bold; the expected proportion given an equal representation of each pattern is ∼11%. n.a., not applicable.*

**FIGURE 1:**
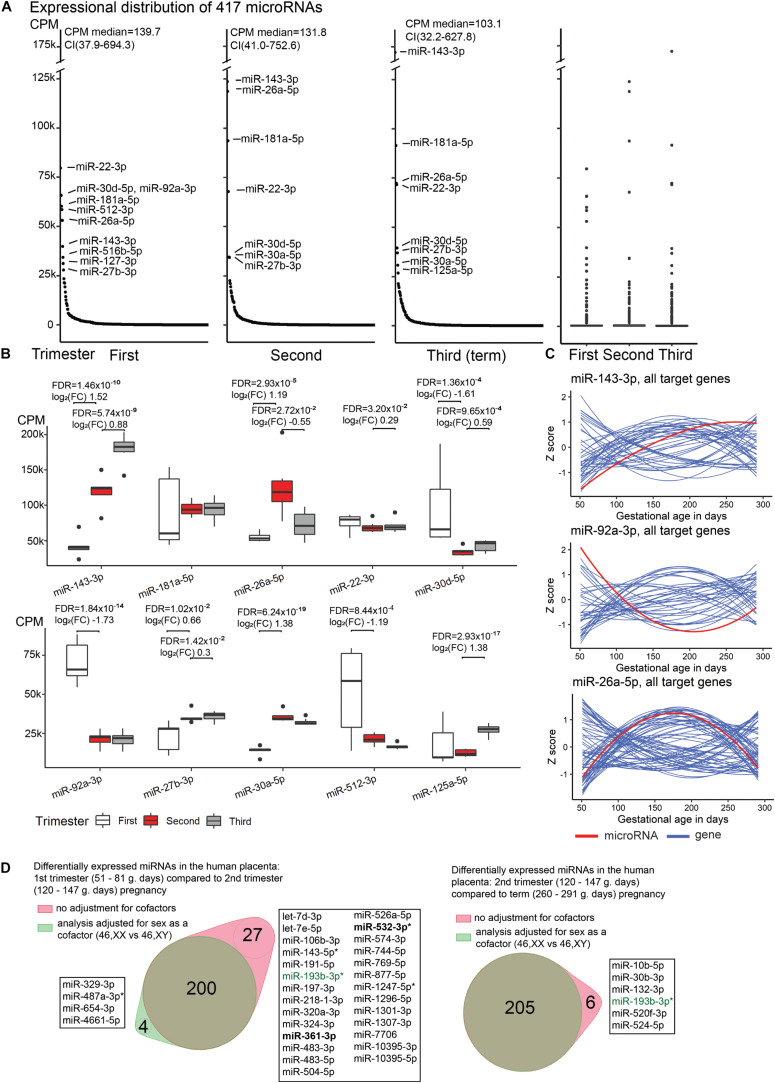
Expressional distribution of placental miRNome. **(A)** Transcript levels of the analyzed 417 miRNAs in the first (median 60; range 51–81 g.days) and second trimester (121; 108–140 g.days) and term placental samples (284; 260–291 g.days). miRNA expression was quantified in counts per million reads mapped (CPM). Highly expressed miRNAs (CPM > 25,000) are indicated. Full details are provided in Supplementary Table 1. **(B)** Trimester-specific expression levels of placental miRNAs with the highest transcript levels. Differential expression testing between the three trimesters of pregnancy was implemented in DESeq2 (ver. 1.22.2) (Love et al., 2014) package for R with default settings. Log_2_(FC), log_2_ fold change in CPM; FDR, false discovery rate, calculated based on Benjamini–Hochberg method. **(C)** Gestational expression dynamics of some most highly transcribed placental miRNAs miR-143-3p, miR-92a-3p, miR-26a-5p compared to the transcript levels of their high-confidence target genes predicted in the miRTarBase (*n* = 47, 39, 74, respectively). Transcriptome data were derived from the published RNA-Seq datasets of the same placental samples representing first (*n* = 5) and second (*n* = 7) trimester, and normal term pregnancy (*n* = 8) (Sõber et al., 2015; Pilvar et al., 2019). miRNA and gene expression levels are presented in *Z*-scores; expression data for miRNA is shown in red and for target genes in blue. **(D)** Differentially expressed placental miRNAs between first (M/F, *n* = 2/3) and second trimester (*n* = 4/3), second trimester and term pregnancy (*n* = 5/3) samples, with or without incorporating fetal sex (46, XY vs. 46, XX) as a cofactor. X-linked miRNAs are highlighted in bold and differentially expressed miRNAs in preeclampsia are indicated with asterisk (*). miR-193-3p (green) showed sex-modulated transcript levels in both comparisons. F, female; M, male.

Placental miRNome in uncomplicated pregnancies was assessed in 20 samples: five cases representing first trimester [median 60 (51–81) gestational days, g.days], seven second trimester [121 (108–140) g.days], and eight term pregnancy [284 (260–291) g.days] cases. Overall, a broad variability in expression levels of individual placental miRNAs were measured in all trimesters (Figure 1A and Supplementary Figure 1). Median expression level of 417 analyzed miRNAs did not differ across the three trimesters of pregnancy (Kruskal–Wallis test, *p* = 0.24). However, a non-significant decreasing trend of median values was observed from 140 CPM (range 1–79,604) in first trimester, to 132 CPM (4–123,631) in second trimester and 103 CPM (7–172,159) in term placental samples.

A handful of miRNAs were identified with extremely high (CPM > 25,000) transcript levels throughout gestation (Figures 1A,B). Among these, miR-143-3p showing gradually increasing transcript levels from early pregnancy until term appeared as a potential marker for pregnancy progression and placental maturation. In total 47 high-confidence target genes for miR-143-3p were identified in the miRTarBase database (Supplementary Table 5). Majority, 93% of them clustered to the Gene Ontology (GO) pathway ‘cellular response to stimulus’ (GO:0051716; FDR = 7.2 × 10^–10^; Supplementary Table 6). More specific enriched (FDR < 0.05) functional categories relevant to pregnancy included, e.g., ‘PI3K-Akt signaling pathway’ (KEGG:04151; 34% of target genes), ‘Endocrine resistance’ (KEGG:01522; 31%), ‘collagen metabolic process’ (GO:0032963; 12%), ‘regulation of glucose transmembrane transport’ (GO:0010827; 12%), ‘growth factor binding’ (GO:0019838; 12%). Other examples of gestational age-dependent highly expressed miRNAs were miR-92a-3p, miR-30d-5p and miR-512-3p (specifically increased in first trimester), miR-26a-5p (second trimester), miR-125a-5p (at term). Some major miRNAs exhibited constant expression throughout gestation (e.g., miR-181a-5p, miR-22-3p).

Comparative assessment of the transcript levels of miR-143-3p, miR-92a-3p and miR-26a-5p with the expression of predicted target genes in the corresponding RNA-Seq dataset revealed a substantial group of mRNA/lincRNAs with reverse expression compared to the miRNA (Figure 1C and Supplementary Table 7). However, significant expressional correlations identified for miR-143-3p and miR-92a-3p (Kendall rank correlation coefficient, *p* < 0.05) included nearly equal proportions of reversely and directly correlated target genes (9/7 and 8/5, respectively; binominal test, *p* > 0.58). There was an under-representation of genes showing tight negative vs. positive correlation with the miR-26a-5p transcript levels (8/21; *p* = 0.024). Interestingly, positively correlated loci represented cancer driver genes (*MYC, PRKCD, RB1, CDK6*), molecules involved in immune response (*MALT1, PIK3CG*), as well as cellular and hormonal signaling (*HGF, PTPN13, IGF1, SMAD1, ESR1, CTGF*). These functional groups are well-known to be involved in placental development and role in supporting the pregnancy.

### Expression Dynamics of miRNAs Is Linked to Placental Development and Function

**FIGURE 2:**
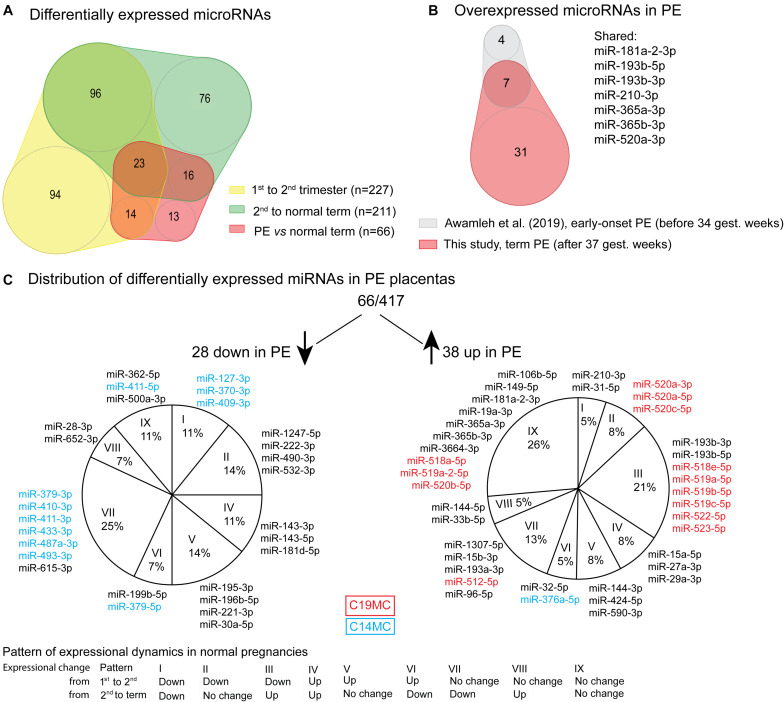
Differentially expressed miRNAs in preeclampsia (PE). **(A)** Overlap of differentially expressed miRNAs in PE (Supplementary Table 21) with miRNAs showing gestational dynamics (Supplementary Tables 8, 9). **(B)** Significantly upregulated miRNAs overlapping between term PE placentas in the current study and early-onset PE placentas in miR-Seq study by Awamleh et al. (2019). **(C)** Distribution of differentially expressed miRNAs in PE placentas according to their gestational dynamics patterns (Table 2B). miRNAs transcribed from placental-specific C14MC and C19MC clusters are highlighted in blue and red color, respectively.

The majority, 319 of 417 (76.5%) of tested miRNAs, exhibited significant gestational expression dynamics (Table 2B and Supplementary Tables 8, 9). In total, 227 (54.4%) miRNAs were differentially expressed between first and second trimester [FDR < 0.05; log_2_(FC) from −4.91 to 2.84; 125 down- and 102 upregulated], and 211 miRNAs (50.1%) between second trimester and term pregnancy placental samples [FDR < 0.05; log_2_(FC) from −2.41 to 2.52; 110 down- and 101 upregulated]. More than a quarter of tested miRNAs (*n* = 119/417; 28.5%) represented DEmiRs in both comparisons, indicating their potential critical contribution in fine-tuning placental transcriptome profile in gestational age-dependent manner until term (Figure 2A and Supplementary Table 10).

The 319 placental miRNAs exhibiting trimester-dependent differential expression were explored for their predicted target genes in the miRTarBase database (Supplementary Table 11). An overrepresentation of target genes for upregulated compared to downregulated miRNAs was observed – 1305 vs. 773 genes in first vs. second trimester and 1207 vs. 888 in second trimester vs. term comparisons (χ^2^-test, *p* = 6.2 × 10^–4^). Only a small fraction of genes (14.1–17.7%; Supplementary Table 11) represented trimester-specific targets of dynamic miRNAs, despite nearly two thirds of DEmiRs (200 of 319) being detected significant increase or decrease only in one trimester (Figure 2A). The shared list of target genes of dynamic miRNAs supported the coordinated action of the placental miRNome in modulating the expression of key placental genes during gestation.

*In silico* functional profiling of genes targeted by placental miRNAs with progressively increasing transcript levels from early pregnancy to term revealed a significant enrichment of GO pathways representing broad basic cellular and tissue functions (FDR < 0.05; Table 3 and Supplementary Tables 12, 13). Examples of modulated processes were ‘protein binding’ (GO:0005515; >90% of target genes) and ‘cytosol’ (GO:0005829; ∼48%), ‘cytoplasmic stress granule (GO:0010494; ∼31% of the pathway genes) and ‘extracellular matrix structural constituent conferring tensile strength’ GO:0030020; 43.5%). Target genes for downregulated miRNAs in second compared to first trimester placentas represent more focused functional groups, possibly supporting the placental role in fine-tuning the fetal development in mid-gestation. These include, e.g., ‘membrane-enclosed lumen’ (GO:0031974; 54.4% of target genes) and ‘cellular response to chemical stimulus’ (GO:0070887; 52.9%); as well as ‘thymocyte apoptotic process’ (GO:0070242; 41.2% of pathway genes), ‘DNA alkylation’ (GO:0006305; 24.4%), ‘PML body’ (GO:0016605; 22.6%), ‘regulation of cell size’ (GO:0008361; 16.5%) and ‘regulation of calcium ion transport’ (GO:0051924; 15.6%). Loci that represent targets for downregulated miRNAs at term cluster in biological pathways relevant to the preparation for the delivery, such as ‘positive regulation of smooth muscle contraction’ (GO:0045987; 42.1% of pathway genes), ‘phosphatidylinositol-3-phosphate biosynthetic process’ (GO:0036092; 39.4%) and ‘regulatory RNA binding’ (GO:0061980; 38.7%).

**TABLE 3:** Top Gene Ontology (GO) pathways of predicted target genes regulated by miRNAs showing gestational dynamics.

Gene Ontology		Pathway genes	Target genes in enrichment query^a^		Enrichment
Term	Name	%	*n*	%	*P*-value^b^
**Target genes for miRNAs significantly UPregulated in transition from first to second trimester^c^**					
GO:0005515	Protein binding	11.5%	991	90.9%	7.5 × 10^–76^
GO:0048522	Positive regulation of cellular process	19.9%	728	66.8%	4.0 × 10^–160^
GO:0005829	Cytosol	12.7%	525	48.2%	1.5 × 10^–25^
GO:0003730	mRNA 3′-UTR binding	32.7%	18	1.7%	9.9 × 10^–4^
GO:0051881	Regulation of mitochondrial membrane potential	28.3%	17	1.6%	2.0 × 10^–2^
GO:0010494	Cytoplasmic stress granule	33.3%	15	1.4%	7.2 × 10^–3^
GO:0030212	Hyaluronan metabolic process	48.1%	13	1.2%	2.2 × 10^–4^
GO:0061980	Regulatory RNA binding	41.9%	13	1.2%	1.6 × 10^–3^
GO:0010586	miRNA metabolic process	40.7%	11	1.0%	1.7 × 10^–2^
GO:0030020	Extracellular matrix structural constituent conferring tensile strength	43.5%	10	0.9%	2.2 × 10^–2^
**Target genes for miRNAs significantly DOWNregulated in transition from first to second trimester^c^**					
GO:0031974	Membrane-enclosed lumen	8.0%	353	54.4%	9.7 × 10^–23^
GO:0070887	Cellular response to chemical stimulus	15.6%	343	52.9%	7.1 × 10^–97^
GO:0072659	Protein localization to plasma membrane	13.1%	26	4.0%	2.6 × 10^–2^
GO:0019867	Outer membrane	13.5%	24	3.7%	3.6 × 10^–2^
GO:0051480	Regulation of cytosolic calcium ion concentration	13.9%	23	3.5%	3.4 × 10^–2^
GO:0051924	Regulation of calcium ion transport	15.6%	22	3.4%	7.5 × 10^–3^
GO:0008361	Regulation of cell size	16.5%	21	3.2%	4.8 × 10^–3^
GO:0016605	PML body	22.6%	19	2.9%	9.1 × 10^–5^
GO:0035296	Regulation of tube diameter	19.0%	15	2.3%	2.6 × 10^–2^
GO:0006305	DNA alkylation	24.4%	11	1.7%	3.3 × 10^–2^
**Target genes for miRNAs significantly UPregulated in transition from second trimester to term^c^**					
GO:0005515	Protein binding	10.6%	909	90.1%	7.1 × 10^–64^
GO:0048518	Positive regulation of biological process	17.0%	694	68.8%	1.1 × 10^–133^
GO:0005829	Cytosol	11.8%	488	48.4%	5.8 × 10^–24^
GO:0046914	Transition metal ion binding	14.5%	109	10.8%	7.8 × 10^–7^
GO:2000060	Positive regulation of ubiquitin-dependent protein catabolic process	22.7%	20	2.0%	4.0 × 10^–2^
GO:0000045	Autophagosome assembly	23.2%	19	1.9%	4.8 × 10^–2^
GO:0010494	Cytoplasmic stress granule	31.1%	14	1.4%	1.7 × 10^–2^
GO:0030020	Extracellular matrix structural constituent conferring tensile strength	43.5%	10	1.0%	1.2 × 10^–2^
GO:1902751	Positive regulation of cell cycle G2/M phase transition	43.5%	10	1.0%	1.2 × 10^–2^
GO:0070242	Thymocyte apoptotic process	52.9%	9	0.9%	4.6 × 10^–3^
**Target genes for miRNAs significantly DOWNregulated in transition from second trimester to term^c^**					
GO:0005515	Protein binding	8.0%	684	91.3%	1.9 × 10^–52^
GO:0048518	Positive regulation of biological process	13.2%	537	71.7%	3.1 × 10^–113^
GO:0043233	Organelle lumen	9.0%	395	52.7%	3.5 × 10^–22^
GO:0019898	Extrinsic component of membrane	14.5%	29	3.9%	2.1 × 10^–2^
GO:0007204	Positive regulation of cytosolic calcium ion concentration	16.1%	25	3.3%	1.4 × 10^–2^
GO:0003730	mRNA 3′-UTR binding	25.5%	14	1.9%	8.4 × 10^–3^
GO:0036092	Phosphatidylinositol-3-phosphate biosynthetic process	39.4%	13	1.7%	6.1 × 10^–5^
GO:0030301	Cholesterol transport	25.0%	13	1.7%	2.4 × 10^–2^
GO:0061980	Regulatory RNA binding	38.7%	12	1.6%	2.7 × 10^–4^
GO:0045987	Positive regulation of smooth muscle contraction	42.1%	8	1.1%	2.0 × 10^–2^

*^a^Top 10 pathways per each gene list analysis are shown, sorted by the highest percentage of representation among the query genes. Full results of in silico functional enrichment analyses of gene lists are in Supplementary Tables 12, 13. ^b^P-value corrected for multiple testing by g: SCS method (Reimand et al., 2007). ^c^Query lists of miRNA target gene were extracted from the miRTarBase database (Huang et al., 2020).*

The tested 417 placental miRNAs were assigned to one of nine subgroups representing their temporal expression dynamics pattern across three trimesters of pregnancy (Table 2B and Supplementary Figure 2). The most prevalent expression dynamics pattern represented miRNAs exhibiting specifically high transcript levels in early pregnancy (*n* = 67 miRNAs, ∼16%). The second frequent pattern reflected miRNAs that were downregulated only at term (*n* = 54, ∼13%). High miRNA expression restricted to second trimester was the rarest observed expressional pattern (*n* = 26, ∼6%). A stable expressional window from early pregnancy to term was identified for 98 miRNAs (23.5%) with 900 predicted target genes (Supplementary Tables 14, 15). Among these, the most significantly enriched biological pathways (FDR < 1.0 × 10^–17^) are also implicated in basic cellular and tissue functions, ‘protein binding’ (GO:0005515; 89.5 of target genes), ‘cellular response to organic substance’ (GO:0071310; 43.8%) and ‘nuclear lumen’ (GO:0031981; 43.5%) (Supplementary Table 16).

### Fetal Sex Is Not a Major Modulator of Placental miRNome

When incorporating sex as a cofactor (46, XX; 46, XY) in differential expression testing between the three gestational trimesters, the list of DEmiRs remained largely the same (Figure 1D and Supplementary Tables 8, 9). In second trimester [(M)ale/(F)emale, *n* = 4/3) compared to normal term placentas (M/F, *n* = 5/3), only six out of 211 (2.8%) DEmiRs were ‘lost’ after correction for fetal sex. The analysis of first (M/F, *n* = 2/3) compared to second trimester pregnancy samples with or without fetal sex as a cofactor resulted in 200 shared miRNAs, whereas four and 27 miRNAs were additionally detected in one sub-analysis. Only a single miRNA, miR-193-3p, was highlighted in both tests comparing trimester-specific miRNomes and may represent a locus with sex-specific expression level. The nature of other minor differences in the identified miRNAs lists cannot be speculated as testing small sample sets may result in spurious outcomes.

To evaluate further the role of fetal sex on placental miRNome, term pregnancy samples representing 46, XY (*n* = 19, 260–291 g.days) vs. 46, XX (*n* = 21, 262–288 g.days) cases were compared (Supplementary Table 17). Statistically significant differential expression was identified for three miRNAs: X-linked miR-361-5p, autosomal miR-378a-3p and miR-130b-3p. Additionally, miR-101-5p showed placental sex-modulated expression when the analysis was adjusted for pregnancy complications. These miRNAs did not overlap with the candidates identified in trimester-specific analysis. Overall, the analysis outcome suggested that fetal sex is not a major modulator of placental miRNome.

### C14MC and C19MC Clusters Have Key Role in Human Placental miRNome and Transcriptome

**FIGURE 3:**
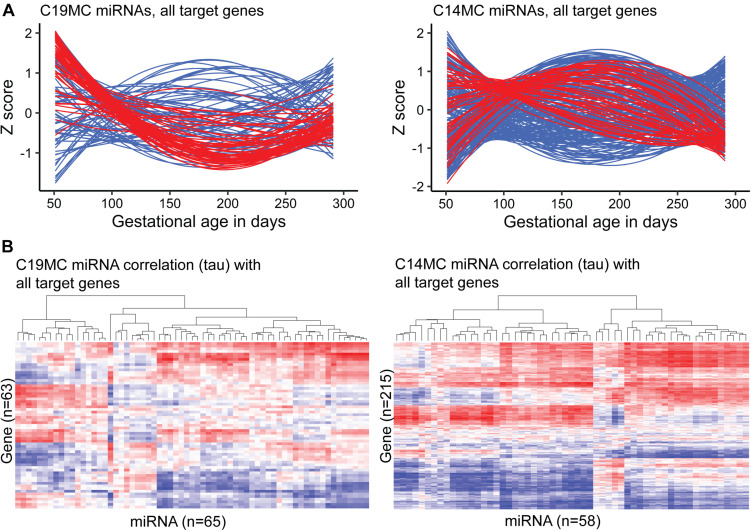
Expression dynamics of C19MC and C14MC miRNA clusters across gestation compared to the transcript levels of their miRTarBase target genes. Placental transcript levels were confidently quantified for 63/76 and 215/262 predicted target genes of C19MC and C14MC, respectively. **(A)** miRNA and gene expression levels during pregnancy presented in *Z*-scores; expression data for miRNAs is shown in red and for target genes in blue. **(B)** Heatmap and hierarchical clustering of miRNA–target gene expression data based on the calculated correlation coefficient Kendall tau.

A notably high fraction, 125 of 417 (∼30%) expressed miRNAs belonged to the primate-specific miRNA cluster C19MC (detected mature placental miRNAs, *n* = 65; 15.6%) or to the eutherian-specific clusters C14MC (*n* = 58; 13.9%) and miR-371–373 (*n* = 2, 0.5%) (Table 2A and Supplementary Table 1). These clusters showed markedly different patterns of gestational expression dynamics. About ∼2/3 of C19MC and the miR-371–373 clusters are specifically highly transcribed in early pregnancy with a significant drop in second trimester and a slight increase at term (Figure 3A, Table 2B, and Supplementary Tables 8, 9). The C14MC cluster showed diverse expression in first trimester, but more coordinated transcript levels in later gestational ages. The majority of C14MC miRNAs showed high expression in second trimester and significant downregulation before term. Only five C14MC, but 22 C19MC miRNAs exhibited stable expression levels from early pregnancy until delivery.

Among confident target genes in miRTarBase database reported for the C19MC and C14MC miRNAs, 63/76 and 215/262 loci were expressed in the current RNA-Seq dataset (Supplementary Tables 18, 19). Indicating the distinct roles of C19MC and C14MC in shaping human placental transcriptome across gestation, only 21 of these target genes overlap. Most target genes are modulated explicitly by either C19MC or C14MC miRNAs. Assessment of gestational expression dynamics of C19MC and C14MC miRNAs and their predicted target genes supports their mutual functional relationship (Figures 3A,B). The major cluster of C19MC miRNAs have the lowest transcript levels at ∼180–200 g.days and a substantial proportion of their predicted target genes show increased expression during mid-gestation. Interestingly, the minor cluster of C19MC miRNAs is strongly positively correlated with over 1/3 of the analyzed genes. The majority of C14MC target genes belong to distinct co-expression groups that form clusters based on either negative or positive transcriptional correlation with miRNAs. Large notable clusters of miRNA–gene pairs showing directly and inversely correlated expression dynamics suggested potential functional relationships in both scenarios.

The top GO terms with significant enrichment of C19MC target genes (FDR < 1.0 × 10^–5^, Supplementary Table 20) were indicative of processes related to transcriptional activation and cellular signaling that are critical in early pregnancy, such as ‘response to organic substance’ (GO:0010033), ‘transcription regulatory region DNA binding’ (GO:0044212), ‘kinase binding’ (GO:0019900). Notably, most significantly enriched KEGG and REAC categories comprise of genes implicated in various cancers or are responsible for signaling pathways of cancer driver genes. The C14MC miRNAs were implicated in more basic cellular functions that are essential throughout pregnancy, such as ‘cellular response to chemical stimulus’ (GO:0070887), ‘protein binding’ (GO:0005515), ‘intracellular organelle lumen’ (GO:0070013). However, some target genes of C14MC reflect specific placental features, such as ‘DNA-methyltransferase activity’ (GO:0009008) potentially linked to epigenetic programming or ‘regulation of cell size’ (GO:0008361) possibly referring to the development of large multinucleated placental cell types.

### Major Shift in Placental miRNome in Preeclampsia and Link to Affected Fetal Growth

**FIGURE 4:**
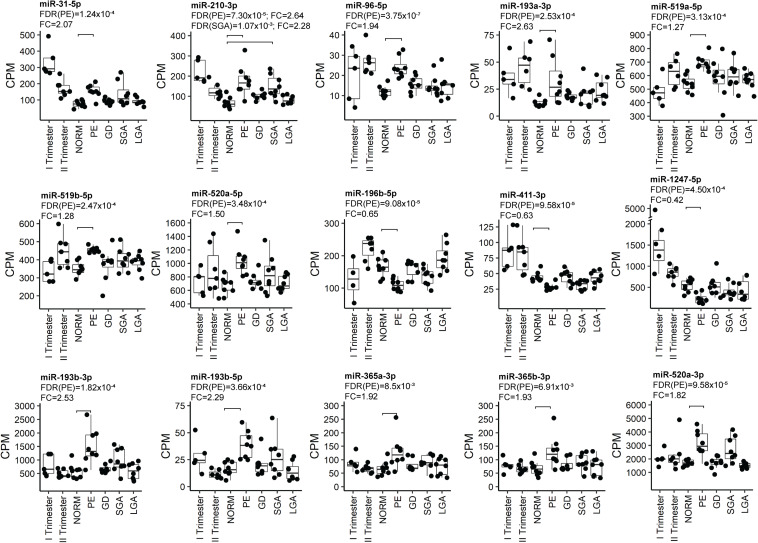
Examples of the most significant differentially expressed miRNAs in PE. miRNA expression was quantified in counts per million mapped reads (CPM). FDR, false discovery rate, calculated based on Benjamini–Hochberg method; GD, gestational diabetes; NORM, normal term pregnancy; PE, preeclampsia; LGA, large-for-gestational-age; FC, fold change in CPM; SGA, small-for-gestational-age.

Placental miRNomes representing term cases of late-onset preeclampsia (LO-PE), gestational diabetes (GD), small- and large-for-gestational-age newborns (SGA, LGA) were tested for differential expression in comparison to uncomplicated pregnancies (*n* = 8 in each group; all cases after 37th g.week; Table 1). Only PE placentas demonstrated a major shift in their miRNome profile that affected 66 of 417 (15.8%) miRNAs (FDR < 0.05; Figure 2 and Supplementary Tables 21, 22). None of these miRNAs was detected to be modulated by fetal sex (Supplementary Table 17). Seven significantly upregulated miRNAs overlapped with the placental DEmiRs reported in early-onset PE cases (EO-PE, before 34th g.week) (Awamleh et al., 2019; Figure 2B). Several of these showed large changes in their expression level: miRNAs miR-210-3p (FC = 2.64), miR-193b-3p (2.53), miR-193b-5p (2.29), miR-365b-3p (1.93), miR-365a-3p (1.92), miR-520a-3p (1.82) (Figure 4 and Supplementary Table 21). Notably, 13 of 38 (34%) upregulated DEmiRs were transcribed from the C19MC cluster and only one from C14MC, whereas 11 of 28 (39%) downregulated miRNAs were transcribed from the C14MC and none from the C19MC cluster (Figure 2C).

Differentially expressed miRNome in PE was comprised of both dynamic and stable miRNAs. No specific pattern of normal gestational dynamics was preferentially altered (Figures 2A,C). Several miRNAs normally downregulated at term were characterized by increased transcript levels in PE placentas corresponding to their typical mid-gestation values (e.g., miR-210-3p, miR-31-5p, miR-96-5p, miR-193a-3p, miR-519a/b-5p; Figure 4). On other occasions, miRNA expression in PE placentas was significantly downregulated compared to other analyzed samples (e.g., miR-196b-5p, miR-411-3p, miR-1247-5p). PE miRNome also showed aberrant upregulation of several miRNAs that are normally stably expressed across gestation (e.g., miR-365a/b-3p).

Six miRNAs with altered transcript levels in LO-PE represented also DEmiRs in term SGA placentas – upregulated miR-210-3p, miR-512-5p, miR-32-5p, miR-19a-3p and miR-590-3p, and downregulated miR-379-5p (Supplementary Table 23). No miRNAs exhibited significant expressional changes in the GD and LGA cases compared to normal term placenta (Supplementary Tables 24,25).

### Differentially Expressed miRNAs in PE Exhibit a Coordinated Effect on the Transcriptome

**FIGURE 5:**
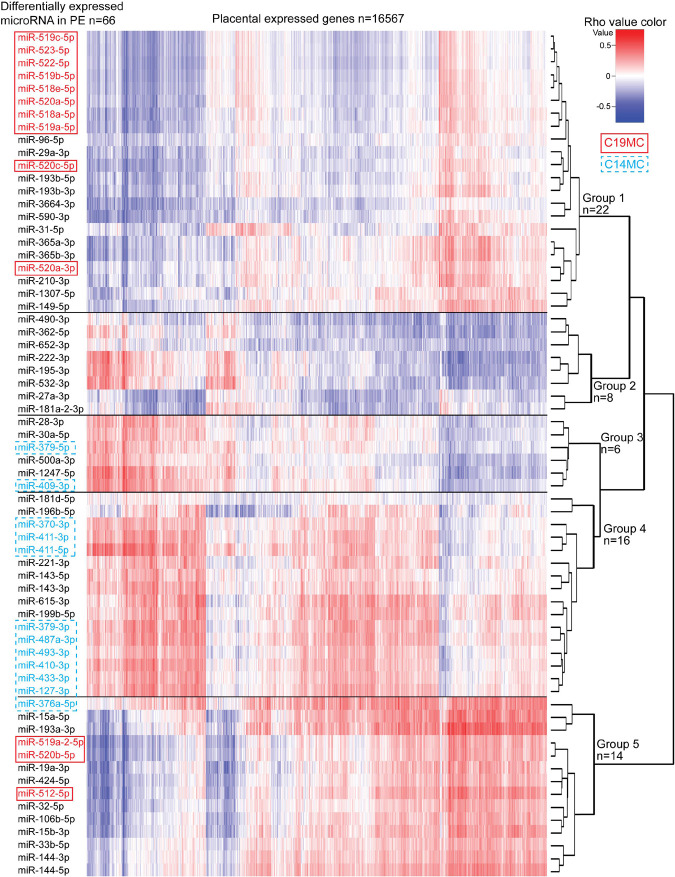
Correlation analysis between miRNAs altered in preeclampsia and the whole transcriptome of 40 term placental samples. Expressional correlation of 66 miRNAs with mRNA transcripts of 16,567 genes (RNA-Seq data from Sõber et al., 2015) was evaluated using Spearman’s correlation coefficient (parameter rho). Spearman’s rho was estimated for 1,093,422 miRNA-gene pairs (Supplementary Data 1). The heatmap shows the hierarchical clustering of miRNAs based on the expressional correlation with mRNA transcripts of coding/lincRNA genes. Each row represents one miRNA and each column one gene. Expressional correlation is presented from –1 (maximum negative correlation, blue color) to 1 (maximum positive correlation, red). The value 0 indicates no correlation. miRNAs groups G1-G5 were formed based on their clustering. More details on genes with correlated expression in each miRNA group G1–G5 are presented in Supplementary Tables 28–32.

Correlation analysis between the expression levels of 66 placental DEmiRs identified in PE and placental transcriptome was performed using the corresponding miR-Seq and RNA-Seq datasets of 40 term pregnancy samples. Hierarchical clustering based on the expressional correlation with the transcript levels of 16,567 genes assigned the tested miRNAs into five groups G1-G5, each containing 6–22 miRNAs (Figure 5 and Supplementary Data 1). In these groups, there was a highly non-random distribution of miRNAs from C19MC (G1:10 miRNAs, G5:3) and C14MC (G4:9, G3:2, G5:1) clusters (χ^2^-test, *p* = 1.5 × 10^–5^), providing further support to their distinct roles in modulating placental transcriptome. The C14MC cluster outlier miRNA that didn’t properly fit in either groups G4 or G5 was miR-376a-5p. Furthermore, this miRNA already stood out in the differential expression analysis with an opposite behavior compared to the rest of the C14MC miRNAs (Figure 2C).

Six of 10 miRNAs with the highest number of negatively correlated placental transcripts belonged to miRNA group G2, e.g., miR-490-3p (median Spearman’s rho across 40 placentas <–0.3, 4375 genes vs. rho > 0.3, 55 genes), miR-27a-3p (2957 vs. 25), miR-362-5p (2823 vs. 427) (Supplementary Tables 22, 26). Among the C19MC miRNAs that are significantly upregulated in PE placentas, the largest proportion of negatively correlated genes was detected for miR-522-5p (1780 negative vs. 148 positive correlations) and miR-518a-5p (1731 vs. 154) clustering to group G1. In general, groups G1 and G2 showed confident negative correlation with a large number of genes (median rho <–0.3 and for all individual samples <–0.1: 342 and 239 genes, respectively; Supplementary Table 27). In contrast, groups G3-G5 were positively correlated with a substantial proportion of the placental transcriptome (median rho > 0.3 and for all individual samples >0.1; 1077, 484, and 2537, respectively). This included also C14MC cluster miRNAs that are significantly downregulated in PE.

Gene enrichment analysis was performed to clarify the potential functional link between the PE-linked miRNAs clustered into groups G1–G5 and their most significantly correlated placental transcripts (Figure 5 and Supplementary Tables 28–32). Group G2 miRNAs appeared as potential key modulators of genes implicated in basic cellular processes, such as organelle function and ribosomal biogenesis, DNA and RNA metabolism, cell cycle and senescence (Table 4). However, expression of miRNAs in groups G1, G3, and G4 was correlated with functionally more specific gene categories, encoding proteins involved in extracellular matrix formation (G1, G4), DNA replication and immunoglobulin complex (G3), synaptic and ion channel activity, adrenergic signaling, renin and insulin secretion (G4).

**TABLE 4:** Functional enrichment of genes showing correlated expression in term placentas with 66 preeclampsia linked DEmiRs.

miRNA group/	Gene Ontology		Pathway genes	Correlated genes in enrichment query		Enrichment
correlation direction^a^	Term	Name	%	n	% n	*P*-value^b^
Group 1/negative^c^	GO:0031012	Extracellular matrix	7.1%	18	7.0%	1.1 × 10^–2^
(258 genes)	GO:0005201	Extracellular matrix structural constituent	10.9%	10	3.9%	4.1 × 10^–2^
Group 2/negative^c^	GO:0043232	Intracellular non-membrane-bounded organelle	2.6%	85	39.5%	2.4 × 10^–2^
(215 genes)	GO:0007049	Cell cycle	3.3%	47	21.9%	1.9 × 10^–2^
	GO:0051301	Cell division	5.0%	25	11.6%	3.0 × 10^–3^
	GO:0042254	Ribosome biogenesis	7.1%	19	8.8%	3.8 × 10^–4^
	GO:0000280	Nuclear division	6.1%	19	8.8%	4.1 × 10^–3^
	GO:0006260	DNA replication	7.4%	18	8.4%	4.3 × 10^–4^
	GO:0071103	DNA conformation change	8.0%	17	7.9%	3.3 × 10^–4^
	GO:0007059	Chromosome segregation	6.8%	17	7.9%	3.2 × 10^–3^
	GO:0006413	Translational initiation	7.7%	14	6.5%	6.4 × 10^–3^
	GO:0044391	Ribosomal subunit	7.3%	13	6.0%	2.8 × 10^–2^
	GO:0003735	Structural constituent of ribosome	8.5%	13	6.0%	5.2 × 10^–3^
	GO:0022626	Cytosolic ribosome	11.4%	12	5.6%	5.3 × 10^–4^
	GO:0006614	SRP-dependent co-translational protein targeting to membrane	12.2%	11	5.1%	8.7 × 10^–4^
Group 3/negative (122 genes)	GO:0006260	DNA replication	4.5%	11	9.0%	4.8 × 10^–2^
Group 3/positive	GO:0042571	Immunoglobulin complex, circulating	100%	5	0.6%	3.2 × 10^–3^
(825 genes)	GO:0030054	Cell junction	10%	93	11.3%	4.0 × 10^–2^
Group 4/positive^c^	GO:0032501	Multicellular organismal process	4.1%	184	47.5%	5.8 × 10^–4^
(387 genes)	GO:0044459	Plasma membrane part	5.4%	88	22.7%	7.5 × 10^–5^
	GO:0007399	Nervous system development	5.0%	78	20.2%	1.4 × 10^–2^
	GO:0045202	Synapse	6.7%	36	9.3%	2.1 × 10^–2^
	GO:0043062	Extracellular structure organization	8.2%	23	5.9%	4.3 × 10^–2^
	GO:0005216	Ion channel activity	9.6%	18	4.7%	4.3 × 10^–2^
	GO:0016849	Phosphorus-oxygen lyase activity	37.5%	6	1.6%	1.1 × 10^–2^
	GO:0009975	Cyclase activity	35.3%	6	1.6%	1.7 × 10^–2^
Group 5/negative	GO:0005886	Plasma membrane	2.6%	80	42.3%	1.0 × 10^–4^
(189 genes)	GO:0048018	Receptor ligand activity	8.3%	13	6.9%	1.5 × 10^–3^
Group 5/positive	GO:0005634	Nucleus	18.7%	1069	49.6%	5.0 × 10^–3^
(2155 genes)	GO:0048519	Negative regulation of biological process	19.5%	726	33.7%	1.9 × 10^–3^
	GO:0003676	Nucleic acid binding	19.9%	645	29.9%	7.8 × 10^–4^
	GO:0002376	Immune system process	21.2%	421	19.5%	1.8 × 10^–4^
	GO:0007017	Microtubule-based process	24.1%	141	6.5%	1.1 × 10^–2^
	GO:0044772	Mitotic cell cycle phase transition	27.4%	121	5.6%	4.5 × 10^–5^
	GO:0060271	Cilium assembly	28.2%	80	3.7%	3.5 × 10^–3^
	GO:0006413	Translational initiation	39.2%	71	3.3%	1.7 × 10^–9^
	GO:0003735	Structural constituent of ribosome	47.1%	72	3.3%	1.1 × 10^–14^
	GO:0007059	Chromosome segregation	27.6%	69	3.2%	3.9 × 10^–2^
	GO:0022626	Cytosolic ribosome	59.0%	62	2.9%	6.0 × 10^–19^
	GO:0000184	Nuclear-transcribed mRNA catabolic process, nonsense-mediated decay	50.4%	59	2.7%	1.9 × 10^–13^
	GO:0006614	SRP-dependent co-translational protein targeting to membrane	60.0%	54	2.5%	1.1 × 10^–16^
	GO:0006261	DNA-dependent DNA replication	32.8%	45	2.1%	1.0 × 10^–2^
	GO:0006334	Nucleosome assembly	42.3%	44	2.0%	2.5 × 10^–6^
	GO:0048525	Negative regulation of viral process	40.0%	26	1.2%	2.3 × 10^–2^
	GO:0045071	Negative regulation of viral genome replication	48.6%	18	0.8%	2.2 × 10^–2^

*^a^miRNA clusters as formed in the correlation analysis between placental miRNome and transcriptome in 40 term pregnancy samples (heatmap, Figure 5). Transcriptome data of the samples utilized for miR-Seq was derived from the published RNA-Seq dataset (Sõber et al., 2015). The number of genes entering correlation testing with the transcript level of 417 miRNAs was 16,567. Number of genes from the RNA-Seq dataset showing correlated expression with the specific miRNA Group are indicated in brackets. Full details are presented in Supplementary Tables 27–32. ^b^P-value corrected for multiple testing by g: SCS method (Reimand et al., 2007). ^c^No functional enrichment pathways were identified for genes showing positive expressional correlation with miRNAs in Groups 1 and 2, and genes showing negative correlation in Group 4.*

Positive expressional correlation between miRNAs in group G5 and thousands of genes may reflect either true functional relationship or alternatively, co-correlated expression of miRNAs and some housekeeping genes through shared upstream regulatory modulators. Notably, ∼50% of them belonged to the functional category ‘nucleus’ (GO:0005634; enrichment *P* = 5.0 × 10^–3^), being responsible for various DNA, chromatin and RNA-related processes. Another large category represented ‘immune system process’ (GO:0002376, 19.5% of query genes, *P* = 1.8 × 10^–4^).

### The Expression of Several Placental MicroRNAs Is Modulated by Specific SNVs, miR-eQTLs

**FIGURE 6:**
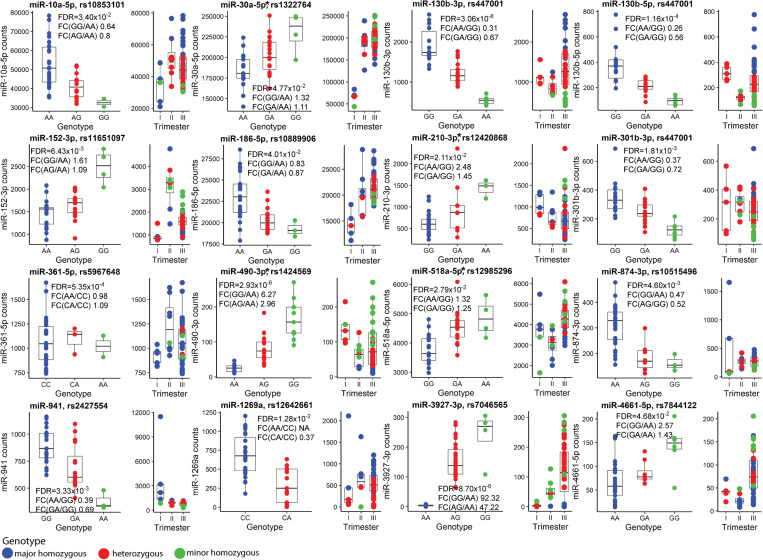
Placental miRNA expression Quantitative Trait Loci (miR-eQTLs). Discovery analysis of single nucleotide variants (SNVs) modulating the expression of flanking miRNAs was carried out in 40 term placental samples and the most significant miR-eQTLs are presented. In each subgraph, miRNA normalized read counts (*y*-axis) are shown for samples stratified based on SNV genotypes (*x*-axis). Blue, red and green colors represent placentas homozygous for the major allele, heterozygous or homozygous for the minor allele of SNV, respectively. Additionally, distribution of genotype-stratified samples in the trimesters are shown. miRNAs that are also differentially expressed in term preeclampsia are indicated with an asterisk (*). Further details are presented in Supplementary Tables 1, 21, 33 and Supplementary Data 1. FDR, false discovery rate; FC, fold change.

Finally, the effect of SNVs on the placental miRNome was investigated. Placental Expression Quantitative Trait Loci (eQTLs) for 417 miRNAs were mapped by genetic association testing between their transcript levels in 40 term placental samples and genotypes of 6,274 common SNVs located ±100 kb from the miRNA genes. In total 66 miR-eQTLs for 16 miRNAs were detected (FDR < 0.05; 3.8% of tested miRNAs; Figure 6, Supplementary Table 33, and Supplementary Data 2). Four of 16 placental miRNAs modulated by eQTLs had also been identified as DEmiRs in PE (miR-30a-5p, miR-210-3p, miR-490-3p, miR-518-5p). Despite the limited sample size, the effect of miR-eQTL on some miRNAs was observed in all three trimesters of pregnancy (e.g., pairs rs447001 and miR-130b-3p/5p, rs2427554 and miR-941, rs12642661 and miR-1269a). The most extreme identified SNV-miRNA pair was rs7046565 (A/G) and miR-3927-3p. The major allele AA-homozygosity completely suppressed the expression of miR-3927-3p. This effect was also detected in second trimester placental samples (Figure 6).

Among 66 identified placental miR-eQTLs, 18 eQTLs were unique to placental miRNAs and 48 have also been reported in the GTEx database as expressional modulators of 53 coding genes (The GTEx Consortium, 2020). In our placental RNA-Seq dataset, 32 of them were expressed. Statistically significant associations (FDR < 0.05) were detected with transcript levels of the *KLHL3* (rs10515496), *SNX11* (rs11651097), *ANO9* and *PTDSS2* (rs12420868) genes (Supplementary Table 34). However, each of these statistical associations were weaker than the originally detected effect on the adjacent miR-874-3p, miR-152-3p, and miR-210-3p, respectively.

### Individual Placental miR-eQTLs Did Not Show a Significant Effect on Term Pregnancy Traits

Three miR-eQTLs (SNVs: rs12420868, rs12985296, rs7046565) showing some nominal associations in the discovery dataset (*n* = 40, Supplementary Table 35) were targeted to replication testing with pregnancy traits in the REPROMETA (*n* = 326) and HAPPY PREGNANCY (*n* = 1,772) pregnancy-related cohorts (Supplementary Table 3 and Supplementary Methods). No statistically significant associations were identified with the height, weight, head and chest circumference of newborns, placental weight and PE or GD incidence in independent cohorts or their meta-analyses (all tests, FDR > 0.05; Supplementary Table 36). A non-significant trend was detected between rs12420868 (eQTL for miR-210-3p) and newborns’ head circumference (meta-analysis: nominal *P* < 0.05; Supplementary Figure 3).

## Discussion

To our knowledge, this investigation represents the most comprehensive multi-omics analysis of human placental miRNome conducted to date. For the first time, this study used miR-Seq to profile simultaneously miRNA expression dynamics across normal gestation from the first trimester to term, and in late-onset pregnancy complications (PE, SGA, LGA and GD). Previous large study integrating placental miRNA and mRNA profiling was focused on a narrow gestational window (∼29–32 g.weeks) and investigated placentas representing idiopathic preterm birth and early-onset PE, intrauterine growth restriction (IUGR) or their combination (Awamleh et al., 2019). Additional innovations in the current study included expressional correlation analysis of miRNA-mRNA transcript pairs across the whole transcriptome and the first report of placental genetic variants that modulate miRNA expression levels.

The study data reveals that the major determinant of placental miRNA transcript levels is gestational age. According to their gestational expression dynamics, placental miRNAs cluster into distinct patterns containing potentially functionally linked miRNAs (Table 3). Only about one quarter of placental miRNAs show constant expression throughout pregnancy. miRNAs with significant gestational dynamics include also those with the highest placental expression (Figures 1A–C). For some top-transcribed miRNAs (miR-143-3p, miR-26a-5p, miR-27b-3p, miR-181a-5p), altered placental and umbilical cord transcript levels have been reported in gestational hypertension, PE and IUGR pregnancies (Hromadnikova et al., 2015, 2017; Muralimanoharan et al., 2016; Huang et al., 2019; Gusar et al., 2020). miR-181a-5p and miR-27b-3p target mRNAs of the *REN* and *ACE* genes, potentially contributing to fine-tuning of the placental renin-angiotensin system (RAS) essential for normal placentation (Arthurs et al., 2019; Huang et al., 2019). Alternations of RAS components have been associated with preterm birth and preeclampsia (Herse et al., 2007; Nonn et al., 2021).

Only limited miRNAs appeared to be potentially modulated by fetal sex (Figure 1D and Supplementary Tables 8, 9, 17). Among these, circulating levels of X-linked miR-361-3p have been reported to differ between obese adolescent females and males (Karere et al., 2021). Interestingly, placental expression of miR-130b-3p was potentially modulated by both, sex and a neighboring SNV, miR-eQTL (Figure 6).

The current multi-dimensional study supported distinct expressional regulation and functional roles of placenta-specific imprinted miRNA clusters C19MC and C14MC in human pregnancy. Paternally expressed young, primate-specific C19MC cluster is highly expressed in early pregnancy, potentially fine-tuning in dosage-sensitive manner the expression level of critical genes that are implicated in deep intrauterine trophoblast invasion and remodeling of uterine spiral arteries at the maternal-fetal interface, enabling unique close contact between maternal and fetal blood streams in humans. Target loci of C19MC include well-established cancer genes (37% of target genes), mediators of various molecular binding, signaling cellular response processes, supporting the suggested co-evolution of genes and processes involved in placentation and mammalian tumorigenesis (Kshitiz et al., 2019). Monoallelic expression of these target genes in early pregnancy may prevent pathological invasiveness of the developing placenta. Interestingly, C19MC miRNAs show a small, but confident increase in their expression level again at term and demonstrate significant upregulation in preeclamptic (PE) placentas (Figures 1D, 2C). It can be speculated that C19MC expression may be upregulated in hypoxic conditions, known to be present in trophoblast development in early pregnancy (Chang et al., 2018), in PE placenta (Redman et al., 2021) and possibly, also in normal term placenta due to its senescence close to parturition (Seno et al., 2018). The maternally expressed C14MC is common for all eutherian mammals. Its high expression level until delivery refers to its role as a ‘guardian’ of normal placental development and unique functions to support the fetus and the mother until delivery. Correlation analysis of miRNA–mRNA expression indicated that C19MC and C14MC miRNAs do not function as uniform miRNA clusters, but contain functional subgroups of miRNAs targeting different sets of genes (Figure 3). Notably, a large fraction of confident target genes of C19MC and especially C14MC show strong positive correlation between their transcript levels and these miRNA clusters. This observation was further extended in the expression correlation analysis of 66 PE-linked miRNAs and the whole placental transcriptome (Figure 5). Positive expression correlation with a large number of confident target genes was also observed for the most highly expressed placental miRNAs (Figure 1). It has been suggested that for several genes, increased mRNA expression has to be followed by activated expression of its regulatory miRNAs in order to maintain the optimal transcript levels (Nunez et al., 2013). In cancer cells, positive miRNA-gene correlations are surprisingly prevalent and consistent across cancer types, and show more distinct patterns than negative correlations (Tan et al., 2019). This is consistent with the data from the current study on the correlated expression patterns of placental miRNome and transcriptome. Coordinated expression of miRNAs and mRNAs could be explained by shared transcription factors. Alternatively, increased levels of some regulatory miRNAs can upregulate a gene by inhibiting its upstream suppressor.

It is well known that aberrant expression of functionally critical miRNAs may lead to a major and potentially pathogenic change in the whole transcriptome. Among the analyzed late pregnancy complications, a major shift in placental miRNome was only observed in PE, but not in GD, SGA or LGA cases (Figure 2A and Supplementary Tables 21, 23–25). The range of expressional alterations of miRNAs in PE placentas was usually not as extensive as the detected gestational age-dependent variation of transcript levels (Figure 4 and Supplementary Tables 1, 8, 9). This outcome was similar to the RNA-Seq based study of late-onset pregnancy complications, whereby only PE, but not GD, SGA or LGA cases showed notable shift in the placental transcriptome (Sõber et al., 2015). None of the miRNAs or their groups stood out a major driver of PE-linked miRNome. More than a third (23 of 66) of miRNAs identified as DEmiRs in PE have been previously described in the context of pregnancy complications or placental function (Supplementary Table 37 and references therein). Interestingly, a similar fraction of placental DEmiRs in PE (24 of 66) have been reported in the context of cancer, especially the miRNAs in group G4 (11 of 16; Figure 5).

Placental miR-Seq has been previously utilized to analyze miRNome in EO-PE (<34 g.week) and/or IUGR (Awamleh et al., 2019). In total 11 of 57 reported DEmiRs (19%) in PE/PE + IUGR (seven miRNAs) or only IUGR (four miRNAs) overlapped with the identified 66 (17%) LO-PE DEmiRs in this study (Supplementary Table 21). Seven miRNAs altered in both EO- and LO-PE represent a robust placental molecular signature of preeclampsia, and also validated by RT-qPCR (Awamleh et al., 2019; Figure 2B). Differently from the limited overlap of affected genes identified in EO-PE and LO-PE placentas in RNA-Seq studies (Sõber et al., 2015; Kaartokallio et al., 2016; Ashar-Patel et al., 2017), placental miRNome appears to undergo a similar expressional shift in both conditions. This observation supports the recently suggested scenario that EO-PE and LO-PE are linked with placental syncytiotrophoblast stress (Redman et al., 2021).

There are several lines of evidence that alterations in placental miRNome contribute to the shared molecular etiology of PE and affected fetal growth. In our dataset, six of 66 PE-altered miRNAs showed also differential expression in SGA cases (Supplementary Table 23). Similarly, six of 57 placental DEmiRs in EO-PE were reported to be shared with EO-PE/IUGR and preterm IUGR conditions (miR-193b-3p, miR-193b-5p, miR-210-3p, miR-520a-3p, miR-365a-3p, miR-365ba-3p) (Awamleh et al., 2019). Additional four PE-linked placental miRNAs in the current study were also reported as DEmiRs in preterm IUGR (miR-193a-3p, miR-376a-5p, miR-500a-3p, miR-362-5p) (Supplementary Table 21).

It is largely unknown whether the active contribution of altered miRNome profile may indeed increase the risk to PE and impaired fetal growth. Studies are needed to clarify whether the observed alterations in placental miRNome represent a passive consequence of the PE and IUGR/SGA condition or a direct response to unfavorable physiological conditions. In this study, we demonstrated that 66 PE-linked miRNAs cluster to five groups with potentially different coordinated impact in modulating the placental transcriptome (Figure 5 and Table 4). Reverse correlation between expression levels of a certain set of miRNAs (Group 2) and a large number of genes may potentially indicate their joint synergetic action in fine-tuning transcript levels of functionally linked loci. The nature of strong positive correlation between groups of miRNAs (Groups 3–4) and transcript levels has to be still determined.

Finally, the study compiled the first list of 66 placental miR-eQTLs, some of which are regulating miRNAs implicated in preeclampsia (Figure 6). The identified miR-eQTLs showed stronger association with the expression of flanking 16 miRNAs rather than 30 neighboring mRNA genes (Supplementary Table 34). It has been proposed that co-localization of GWAS and eQTL signals may uncover the role of gene expression modulating variants (Hormozdiari et al., 2016). So far, only two of the identified placental miR-eQTLs have been reported in the GWAS catalog: rs10853101 (miR-10a-5p) associated with the risk to diverticular disease and rs1424569 (hsa-miR-490-3p) with cardiac PR interval. In perspective, the phenotype effects of placental miR-eQTLs individually or as a component in polygenic risk scores for pregnancy-related traits (Moen et al., 2018; Ursini et al., 2018; Lamri et al., 2020; Steinthorsdottir et al., 2020) are still to be clarified. The pilot association testing of miR-eQTLs with newborn traits in the current study may have suffered from inadequate statistical power due to highly polygenic contribution (each variant with small effect), as well as combined effect of maternal and fetal genetics in modulating anthropometric parameters at birth (Beaumont et al., 2020).

Also the limitations in this study have to be acknowledged. As a robust differential expression analysis was aimed, low-expressed miRNAs that may give spurious results were filtered out. The applied criterion ‘median raw read counts >50 across all 52 samples’ may have excluded some first or second trimester specific miRNAs due to underrepresentation of these samples in the total set. Another limitation was low sample size of first and second trimester placentas, restricting miRNA-mRNA expressional correlation analysis and miR-eQTL association testing to term samples.

In summary, the current study reported a large, multilayered and thorough investigation of placental miRNome and its synergy with placental transcriptome during pregnancy, as well as in normal and complicated term deliveries. The dataset represents a rich catalog for further in-depth studies of specific placental miRNAs, their contribution to shaping normal and pathological placental transcriptome and consequently, their individual and joint functional impact to the pregnancy course. Several highlighted miRNAs represent potential biomarkers for pregnancy monitoring and in perspective, possible targets to even prevent or treat gestational complications.

## Data Availability Statement

The datasets presented in this study can be found in online repositories. The names of the repository/repositories and accession number(s) can be found below: European Genome-phenome Archive (EGA, https://www.ebi.ac.uk/), Accession EGAS00001005378.

## Ethics Statement

The studies involving human participants were reviewed and approved by The Ethics Review Committee of Human Research of the University of Tartu, Estonia (Permissions Nos 146/18, 27.02.2006; 150/33, 18.06.2006; 158/80, 26.03.2007; 221/T-6, 17.12.2012; and 286/M-18, 15.10.2018). The patients/participants provided their written informed consent to participate in this study.

## Author Contributions

ML contributed to the conception and the provision of study materials. RI and ML contributed to the design. RI, TK, and KL contributed to the experimental conduct. RI contributed to the data analysis. RI, KL, TK, and ML contributed to the data interpretation. RI and ML contributed to the manuscript writing. RI, TK, KL, and ML contributed to the critical reading and commenting of the article, and final approval of manuscript. All authors contributed to the article and approved the submitted version.

## Conflict of Interest

The authors declare that the research was conducted in the absence of any commercial or financial relationships that could be construed as a potential conflict of interest.
