# Supplementary material for: Coordinated Expressional Landscape of the Human Placental miRNome and Transcriptome
Source: Front Cell Dev Biol. 2021 Jul 21;9:697947. doi: 10.3389/fcell.2021.697947 (PMC8334369; doi:10.3389/fcell.2021.697947)
Supplement: Supplementary file 1 [file Data_Sheet_1.pdf]

List of Supplementary materials

Supplementary Methods

Supplementary Figure S1. Top 20 placental microRNAs with the highest expression level in the 1st and 2nd trimester of pregnancy, or at term.

Supplementary Figure S2. Distribution of 417 analyzed placental microRNAs according to the expressional dynamics patterns I-IX across pregnancy.

Supplementary Figure S3. Association testing between placental miR-eQTLs and newborn traits: results with nominal p-values <0.05 in REPROMETA, HAPPY PREGNANCY or their combined meta-analysis.

References for Supplementary Methods and Figures

Supplementary Data S1 (separate Data1 .xlsx file). Expression correlation (Spearman rho values) between 66 preeclampsia-associated miRNAs and placental transcripts (n=16,567) in term pregnancy placentas (n=40). This dataset was the input for **Figure 5**.

Supplementary Data S2 (separate Data2.xlsx file). Genome-wide miR-eQTL testing results. The genomic regions flanking the analyzed 417 miRNAs included 6,274 common SNVs (MAF>0.1). In total, 17,302 linear regression association tests were carried out between SNV genotypes and miRNA expression levels, quantified as normalized miRNA read counts. All tests with miR-eQTLs were implemented in PLINK v1.07 using fetal sex and gestational age as cofactors(Purcell et al., 2007). The results were corrected for multiple testing using the Benjamini-Hochberg method, with cut-off FDR <0.05. All of the miR-eQTLs were tested for Hardy-Weinberg equilibrium (**Table S2**).

Supplementary Tables (separate Supplementary_Tables.xlsx file):
Table S1. Median normalized read counts of 417 microRNAs in the analyzed 52 human placental samples representing early, mid- or term pregnancy.
Table S2. Testing genotypes of miR-eQTLs for Hardy-Weinberg equilibrium.
Table S3. Clinical characteristics of the REPROMETA and HAPPY PREGNANCY cohorts.
Table S4. Taqman assays for genotyping (Applied Biosystems, Foster City, USA).
Table S5. Target genes of miR-143-3p, miR-92a-3p, miR-26a-5p based on the miRTarBase database.
Table S6. *In silico* functional enrichment analysis of biological pathways of 47 target genes for miR-143-3p**.**
Table S7. Correlation of the expression of miR-26a-5p, miR-143-3p and miR-92a-3p and their predicted target genes across gestation.
Table S8. Differential expression testing results between the first (n=5) and second (n=7) trimester of pregnancy placental miRNomes (417 tested miRNAs).
Table S9. Differential expression testing results between the second trimester (n=7) and normal term pregnancy (n=8) placental miRNomes (417 tested miRNAs).
Table S10. Differentially expressed microRNAs in pregnancy, stratified as up- or downregulated compared to previous trimester.

Table S11. Differential expression testing results between females (n=21) and males (n=19) of term pregnancy placental miRNomes (417 tested miRNAs).
Table S12. *In silico* functional enrichment analysis of biological pathways of target genes for microRNAs showing differential expression between the 1st and 2nd trimester of pregnancy.
Table S13. *In silico* functional enrichment analysis of biological pathways of target genes for microRNAs showing differential expression between the 2nd trimester and normal term pregnancy.
Table S14. Analyzed microRNAs showing stable expression throughout the three trimesters of pregnancy.
Table S15. Target genes for stably expressed microRNAs between the 1st and 2nd trimester, as well as between 2nd trimester and term pregnancy.
Table S16. *In silico* functional enrichment analysis of biological pathways of target genes for microRNAs showing stable expression throughout pregnancy.
Table S17. Differential expression testing results between 46,XX (n=21) and 46,XY (n=19) placental miRNomes of term pregnancy (417 tested miRNAs).
Table S18. Target genes for stably expressed microRNAs transcribed from the C19MC and C14MC clusters.
Table S19. Correlation of the expression of microRNAs belonging to C19MC or C14MC miRNA clusters and their predicted target genes across gestation.
Table S20. *In silico* functional enrichment analysis of biological pathways of target genes for microRNAs transcribed from C19MC and C14MC.
Table S21. Differential expression testing between placental miRNomes representing preeclampsia and normal term pregnancy cases.
Table S22. Characteristics of 66 microRNAs that exhibited differential expression in preeclamptic placentas.
Table S23. Differential expression testing between placental miRNomes representing pregnancies delivering a small-for-gestational-age newborn and normal term cases.
Table S24. Differential expression testing between placental miRNomes representing pregnancies delivering a large-for-gestational-age newborn and normal term cases.
Table S25. Differential expression testing between placental miRNomes representing pregnancies with gestational diabetes and normal term cases.
Table 26. Placental preeclampsia-linked DEmiRs that are negatively correlated with 10% of the transcriptome.
Table S27. Genes showing expressional correlation with microRNAs identified as differentially expressed in term preeclampsia.
Table S28. *In silico* functional enrichment analysis of biological pathways of genes showing correlated expression with 22 miRNAs clustering to group G1 in miRNA/mRNA correlation matrix.
Table S29. *In silico* functional enrichment analysis of biological pathways of genes showing correlated expression with 8 miRNAs clustering to group G2 in miRNA/mRNA correlation matrix.
Table S30. *In silico* functional enrichment analysis of biological pathways of genes showing correlated expression with 6 miRNAs clustering to group G3 in miRNA/mRNA correlation matrix.
Table S31. *In silico* functional enrichment analysis of biological pathways of genes showing correlated expression with 16 miRNAs clustering to group G4 in miRNA/mRNA correlation matrix.

Table S32. *In silico* functional enrichment analysis of biological pathways of genes showing correlated expression with 14 miRNAs clustering to group G5 in miRNA/mRNA correlation matrix.
Table S33. Statistically significant placental miR-eQTLs identified in association testing between expression levels of 417 placental microRNA and flanking SNVs.
Table S34. Characteristics and literature data of the identified miR-eQTLs.
Table S35. Discovery genetic association testing between miR-eQTLs and pregnancy traits.
Table S36. Replication association testing between three selected miR-eQTLs and term pregnancy traits in the REPROMETA and HAPPY PREGNANCY datasets.
Table S37. Literature data of 66 microRNA exhibiting placental differential expression in late-onset preeclampsia.

Supplementary Methods

Collection and processing of placental sample sets: Placental sampling has recently been described in detail (Kasak et al., 2015; Sõber et al., 2015; Reiman et al., 2017; Kikas et al., 2019). The placentas collected at term or during 2nd trimester medically induced abortion due to maternal medical risks were stored at +4°C after vaginal delivery or C-section, and processed further within one hour. A full-thickness block of 2x2 cm was taken from the middle region of each placenta quarter. The excision site was chosen away from the umbilical cord insertion site, large vessels, and any visible or palpable infarction, hematoma or damage. Samples were washed with 1x Phosphate Buffered Saline (PBS) to remove maternal blood and divided for both DNA and RNA extraction at a later date. The block was further longitudinally dissected through all layers. Sections of 1 g or 100 mg of tissue for RNA extraction were placed into 10 ml or 1 ml RNAlater solution (AM7021; Thermo Fisher Scientific). Samples were kept at +4°C for 1-3 days in RNAlater and then stored at -80°C until RNA extraction. Placental samples used for DNA extraction were placed in dry cryovial immediately after excision and washed with 1x PBS. Placental DNA was extracted using NucleoSpin Tissue kit (Macherey-Nagel, Germany) according to the manufacturer's instruction. All samples were collected by the same medical personnel.

Samples of the 1st trimester placentas were obtained immediately after elective (surgical) termination of pregnancy. The procedure to purify placental chorionic villi is described previously by Sõber et al., 2016 and Kasak et al., 2017. Samples were washed with a solution containing 15 ml Dulbecco's PBS, 0.3 ml penicillin-streptomycin solution 10000U/10000µg/ml and 2 drops of heparin (5000U/ml). Maternal cells were removed under a stereomicroscope (Discovery V8, Zeiss) and chorionic villi containing both cytotrophoblast and syncytiotrophoblast cells of fetal origin were placed into a dry tube (for DNA analysis) or RNAlater solution (for RNA analysis) and stored at -80°C. For the 1st trimester samples, karyotyping was applied to confirm normal male or female karyotype.

RNA and DNA extraction protocols: All placental samples had been kept at -80°C until DNA/RNA isolation. For total RNA isolation, a tissue sample was thawed on ice and a 200–300 mg slice (mostly comprised of chorionic villi) was cut from the middle of the sample. Total RNA was extracted from 200-300 mg of homogenized placental tissue using TRIzol reagent (Invitrogen, Life Technologies). Samples were purified with RNeasy MinElute Columns (Qiagen, Netherlands) when used for Total RNA sequencing. Purity level and concentration of isolated total RNA was measured using NanoDrop® ND-1000 UV-Vis spectrophotometer (Thermo Fisher Scientific Inc., USA) and RIN (RNA integrity number) was estimated using Agilent 2100 Bioanalyzer (Agilent Technologies, USA). DNA was extracted using NucleoSpin Tissue kit (Macherey-Nagel, Germany) according to the manufacturer's instructions.

Total RNA sequencing (RNA-Seq) and data processing: Placental RNA-Seq of the samples analyzed in the current study has been described in detail elsewhere (Sõber et al., 2015; Reiman et al., 2017). The RNA-Seq library preparation, RNA sequencing and raw data processing were conducted at the FIMM Sequencing Laboratory, University of Helsinki, Finland. High-quality DNA-free total RNA (5 µg) was used for rRNA depletion (Ribo-Zero™ rRNA Removal Kit, Epicentre) followed by cDNA synthesis (Life Technologies). Library preparation was performed using the Nextera® Technology (Illumina). Total RNA sequencing was performed on Illumina HiSeq2000 with 46 bp paired-end reads (101 bp for two samples).

The total RNA-Seq datasets were processed using an up-to-date bioinformatics pipeline (Kumar et al., 2017) at the FIMM NGS bioinformatics unit, University of Helsinki. Briefly, the raw reads were corrected for low quality, Illumina adapters, and short length using Trimmomatic (Bolger et al., 2014) with the following parameters: leading: 3, trailing: 3, sliding window: 4:15, illuminaclip 2:30:10, and minlen: 36. Quality trimmed paired-end reads were then aligned by STAR (Dobin et al., 2013) to the human reference genome (GRCh38) with the guidance of EnsEMBL v82 reference gene models. Default 2-pass per-sample parameters were used, except that the overhang on each side of the splice junctions was set to 99. Picard tools (http://picard.sourceforge.net) were used to sort and mark duplicate reads. Reads were assigned to genomic features using SubRead (Liao et al., 2013). Default parameters were used, except that in the feature summations reads were allowed to be assigned to overlapping genome features. Quality control analysis was performed using the RNA-SeQC (Deluca et al., 2012) and FastQC (Andrews, 2010) with default settings and supported by the GRCh38 associated references files.

Genome-wide genotyping of placental samples: Genome-wide genotyping of placental samples investigated for the miRNome profile in this study was performed using Illumina HumanOmniExpress-12-v1 BeadChip (>733,000 SNPs, median spacing 2.1 kb)(Kasak et al., 2015; Kikas et al., 2019; Pilvar et al., 2019). Samples were genotyped with an average overall call rate >99% per individual per genotype. Normalized signal intensity data were obtained through Illumina GenomeStudio software. SNPs were excluded if they deviated from Hardy-Weinberg Equilibrium (HWE; $P<1\times10^{-6}$) or had no minor allele carriers in our dataset. After data filtering, 661,354 genotyped SNPs were taken forward to the next step.

REPROMETA and HAPPY PREGNANCY cohort samples for genetic association testing between miR-eQTLs and pregnancy traits: The study utilized samples from Estonian REPROMETA (full study name "REPROgrammed fetal and/or maternal METAbolism"; recruitment 2006–2011) and HAPPY PREGNANCY (full: "Development of novel non-invasive biomarkers for fertility and healthy pregnancy"; 2013-2015) data sets. Both studies were approved by the Ethics Review Committee of Human Research of the University of Tartu, Estonia (Permissions No 146/18, 27.02.2006; 150/33, 18.06.2006; 158/80, 26.03.2007; 221/T-6, 17.12.2012; 286/M-18, 15.10.2018). All study participants were recruited, and the study material was collected at the Women's Clinic of Tartu University Hospital, Estonia. Written informed consent to participate in the study was obtained from each individual prior to recruitment. The study was carried out in compliance with the Helsinki Declaration and all methods were carried out in accordance with approved guidelines. All participants were of white European ancestry and living in Estonia.

The REPROMETA study recruited family duos or trios (mother, father and placenta) (n=366 families) before or shortly after delivery of a singleton newborn (**Table S3**). The study was designed to include well-defined, clinically diagnosed diverse scenarios of pregnancy outcomes at term (36-42 gestational weeks). The recruited cases represented five clinical subgroups: delivery of a small-for-gestational-age (SGA, birth weight <10th centile) or large-for-gestational-age newborn (LGA, >90th centile), cases of maternal gestational diabetes (GD) or late-onset preeclampsia (PE), and normal term pregnancies (birth weight 10th-90th centile). Maternal-newborn clinical and epidemiologic data were documented retrospectively from self-reported questionnaires and medical records, the collected biological material included placental tissue and parental blood samples. Cases with documented fetal anomalies, chromosomal abnormalities, families with a history of inherited diseases, and patients with known pre-existing diabetes mellitus, chronic hypertension, and chronic renal disease were excluded.

The HAPPY PREGNANCY study prospectively recruited a cohort of 2,334 pregnant women during their first antenatal visit without any pre-selection criteria (**Table S3**). Longitudinal clinical data for the course and pregnancy outcome were extracted from medical records; epidemiological data including reproductive history, parental lifestyle were collected from three questionnaires across gestation.

In both studies, SGA and LGA newborns were defined based on the Estonian Medical Birth Registry (Sildver et al., 2015). The diagnosis of PE and GD followed the international guidelines at the time of recruitment (Metzger, 2010; American College of Obstetricians and Task Force on Hypertension in Pregnancy, 2013). All PE cases represented the severe form of preeclampsia, diagnosed as extreme hypertension (systolic blood pressure ≥160 mmHg and/or diastolic blood pressure ≥110 mmHg), combined with proteinuria of ≥5 g in 24 hours or neurological symptoms. GD was diagnosed when a 75 g oral glucose tolerance test (OGTT) performed at 24–28 weeks of gestation revealed either a fasting venous plasma glucose level of ≥5.1 mmol/l and/or at 1h and 2h later plasma glucose level of ≥10.0 mmol/l and ≥8.5 mmol/l glucose, respectively.

TaqMan qPCR: TaqMan genotyping was performed for the REPROMETA (n=326) and HAPPY PREGNANCY (n=1,772) study cases with available placental tissue. All genotyping reactions were performed in 384 micro-well plates in ViiA 7 Real-Time PCR System (Applied Biosystems, Life Technologies). TaqMan probes were used for rs12985296, rs7046565 and rs12420868, experimental conditions as recommended by the protocol (Applied Biosystems, Life Technologies; **Table S4**). Genotype was called using ViiA 7 QuantStudio Real-Time PCR software (Applied Biosystems, Life Technologies).

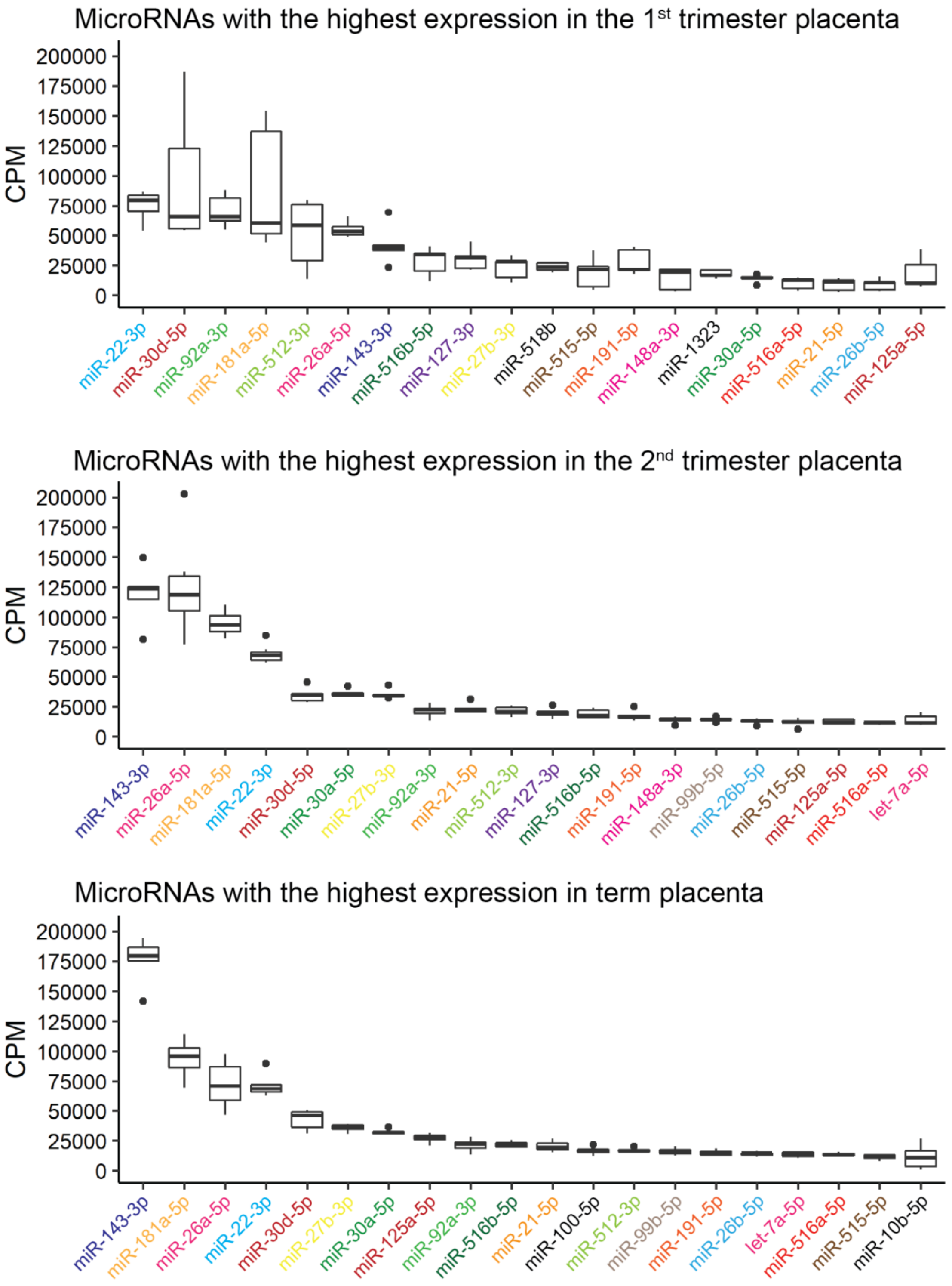

Figure S1. Top 20 placental microRNAs with the highest expression level in the 1st and 2nd trimester of pregnancy, or at term. miRNAs are color-coded to facilitate easier identification of the corresponding data in each subfigure. CPM, counts per million reads mapped; miR, microRNA.

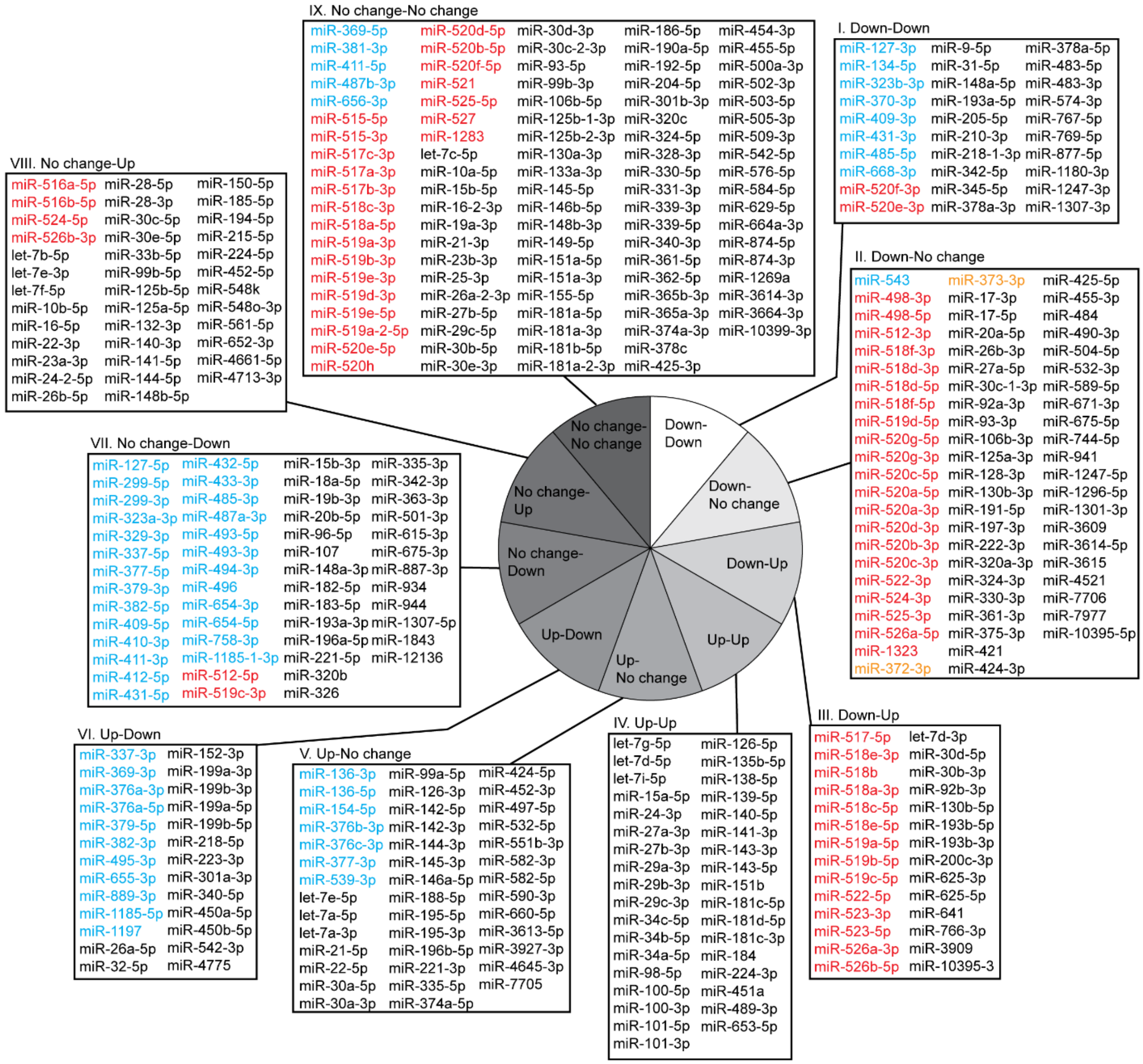

Figure S2. Distribution of 417 analyzed placental microRNAs according to the expressional dynamics patterns I-IX across pregnancy. Each microRNA was assigned to one of the nine patterns of expressional change based on the differential expression testing results between the miRNomes of 1st and 2nd trimester (Up, Down or No change) and 2nd trimester and term placenta (Up, Down or No change) (**Table 2**). For example, 'No change-Up' refers to miRNAs that do not show differential expression between 1st and 2nd trimester, but are significantly up-regulated in term placentas. Normalized expression values of each individual miRNA in the 1st trimester, 2nd trimester and term placentas are provided in **Table S1** and differential expression testing results between trimesters are shown in **Tables S7-S8.**

Red, blue and yellow fonts refer to miRNAs belonging to the placenta-specific miRNA clusters C19MC, C14MC and miR-371–373, respectively.

Chest circumference
rs12420868 (miR-210-3p)

Study	Beta (cm)	CI	P-value
RM	-0.23	-1.25 - 0.8	0.66
HP	-1.23	-2.41 - -0.04	0.04
Meta	-0.66	-1.43 - 0.11	0.09

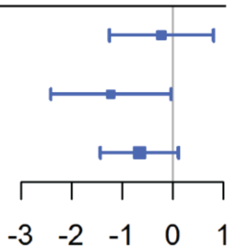

Head circumference
rs12420868 (miR-210-3p)

Study	Beta (cm)	CI	P-value
RM	-0.53	-1.33 - 0.27	0.19
HP	-1.02	-2.22 - 0.18	0.1
Meta	-0.68	-1.35 - -0.02	0.04

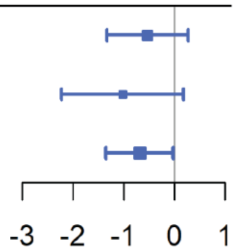

Newborn weight
rs12985296 (miR-518a-5p)

Study	Beta (g)	CI	P-value
RM	-156.6	-272.2 - -41.03	0.01
HP	-18.42	-49.97 - 13.13	0.25
Meta	-28.01	-58.45 - 2.43	0.07

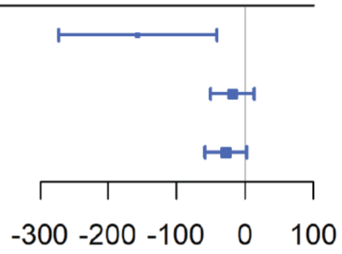

Figure S3. Association testing between placental miR-eQTLs and newborn traits: results with nominal *p*-values <0.05 in REPROMETA, HAPPY PREGNANCY or their combined meta-analysis. microRNA expression Quantitative Trait Locus (miR-eQTL) represents an SNV that modulates the expression level of the flanking miRNA (indicated in brackets). Association testing was carried out between placental genotypes of the REPROMETA (RM, n=326) and HAPPY PREGNANCY (HP, n=1,772) study samples. All tests were performed in PLINK ver. 1.07 (Purcell et al., 2007). Meta-analysis of RM and HP datasets (n=2,097) was implemented in R package meta (ver. 4.15-1)(Balduzzi et al., 2019), under fixed-effect model. CI, confidence interval; Beta, effect of the SNV expressed in the unit of the measurement.

References

American College of Obstetricians, and Task Force on Hypertension in Pregnancy (2013). Hypertension in Pregnancy. *Obstet. Gynecol.* 122, 1122–1131. doi:10.1097/01.AOG.0000437382.03963.88.

Andrews, S. (2010). FastQC: A Quality Control Tool for High Throughput Sequence Data. Available at: http://www.bioinformatics.babraham.ac.uk/projects/fastqc/.

Balduzzi, S., Rücker, G., and Schwarzer, G. (2019). How to perform a meta-analysis with R: A practical tutorial. *Evid. Based. Ment. Health* 22, 153–160. doi:10.1136/ebmental-2019-300117.

Bolger, A. M., Lohse, M., and Usadel, B. (2014). Trimmomatic: A flexible trimmer for Illumina sequence data. *Bioinformatics*. doi:10.1093/bioinformatics/btu170.

Deluca, D. S., Levin, J. Z., Sivachenko, A., Fennell, T., Nazaire, M. D., Williams, C., et al. (2012). RNA-SeQC: RNA-seq metrics for quality control and process optimization. *Bioinformatics*. doi:10.1093/bioinformatics/bts196.

Dobin, A., Davis, C. A., Schlesinger, F., Drenkow, J., Zaleski, C., Jha, S., et al. (2013). STAR: Ultrafast universal RNA-seq aligner. *Bioinformatics*. doi:10.1093/bioinformatics/bts635.

Kasak, L., Rull, K., Sõber, S., and Laan, M. (2017). Copy number variation profile in the placental and parental genomes of recurrent pregnancy loss families. *Sci. Rep.* 7. doi:10.1038/srep45327.

Kasak, L., Rull, K., Vaas, P., Teesalu, P., and Laan, M. (2015). Extensive load of somatic CNVs in the human placenta. *Sci. Rep.* 5, 8342. doi:10.1038/srep08342.

Kikas, T., Rull, K., Beaumont, R. N., Freathy, R. M., and Laan, M. (2019). The Effect of Genetic Variation on the Placental Transcriptome in Humans. *Front. Genet.* 10, 550. doi:10.3389/FGENE.2019.00550.

Kumar, A., Kankainen, M., Parsons, A., Kallioniemi, O., Mattila, P., and Heckman, C. A. (2017). The impact of RNA sequence library construction protocols on transcriptomic profiling of leukemia. *BMC Genomics*. doi:10.1186/s12864-017-4039-1.

Liao, Y., Smyth, G. K., and Shi, W. (2013). The Subread aligner: Fast, accurate and scalable read mapping by seed-and-vote. *Nucleic Acids Res.* doi:10.1093/nar/gkt214.

Metzger, B. E. (2010). International Association of Diabetes and Pregnancy Study Groups recommendations on the diagnosis and classification of hyperglycemia in pregnancy. *Diabetes Care* 33, 676–682. doi:10.2337/dc09-1848.

Pilvar, D., Reiman, M., Pilvar, A., and Laan, M. (2019). Parent-of-origin-specific allelic expression in the human placenta is limited to established imprinted loci and it is stably maintained across pregnancy. *Clin. Epigenetics* 11, 94. doi:10.1186/s13148-019-0692-3.

Purcell, S., Neale, B., Todd-Brown, K., Thomas, L., Ferreira, M. A. R., Bender, D., et al. (2007). PLINK: a tool set for whole-genome association and population-based linkage analyses. *Am. J. Hum. Genet.* 81, 559–75. doi:10.1086/519795.

Reiman, M., Laan, M., Rull, K., and Sõber, S. (2017). Effects of RNA integrity on transcript quantification by total RNA sequencing of clinically collected human placental samples. *FASEB J.* doi:10.1096/fj.201601031RR.

Sildver, K., Veerus, P., and Lang, K. (2015). Sünnikaalukõverad Eestis ja sünnikaalu mõjutavad tegurid: registripõhine uuring. *Eesti Arst - Eesti Arstide Liidu ajakir*, 465–470. Available at: https://eestiarst.ee/sunnikaalukoverad-eestis-ja-sunnikaalu-mojutavad-tegurid-registripohine-uuring/ [Accessed April 20, 2020].

Sõber, S., Reiman, M., Kikas, T., Rull, K., Inno, R., Vaas, P., et al. (2015). Extensive shift in placental transcriptome profile in preeclampsia and placental origin of adverse pregnancy outcomes. *Sci. Rep.* 5, 13336. doi:10.1038/srep13336.

Sõber, S., Rull, K., Reiman, M., Ilisson, P., Mattila, P., and Laan, M. (2016). RNA sequencing of chorionic villi from recurrent pregnancy loss patients reveals impaired function of basic nuclear and cellular machinery. *Sci. Rep.* 6, 38439. doi:10.1038/srep38439.
